# PHGDH is required for germinal center formation and is a therapeutic target in *MYC-*driven lymphoma

**DOI:** 10.1172/JCI153436

**Published:** 2022-05-02

**Authors:** Annalisa D’Avola, Nathalie Legrave, Mylène Tajan, Probir Chakravarty, Ryan L. Shearer, Hamish W. King, Katarina Kluckova, Eric C. Cheung, Andrew J. Clear, Arief S. Gunawan, Lingling Zhang, Louisa K. James, James I. MacRae, John G. Gribben, Dinis P. Calado, Karen H. Vousden, John C. Riches

**Affiliations:** 1 The Francis Crick Institute, London, United Kingdom.; 2 Centre for Haemato-Oncology, Barts Cancer Institute, and; 3 Centre for Immunobiology, Blizard Institute, Queen Mary University of London, London, United Kingdom.; 4 Metabolomics Science Technology Platform, The Francis Crick Institute, London, United Kingdom.

**Keywords:** Immunology, Metabolism, Amino acid metabolism, Immunoglobulins, Lymphomas

## Abstract

The synthesis of serine from glucose is a key metabolic pathway supporting cellular proliferation in healthy and malignant cells. Despite this, the role that this aspect of metabolism plays in germinal center biology and pathology is not known. Here, we performed a comprehensive characterization of the role of the serine synthesis pathway in germinal center B cells and lymphomas derived from these cells. We demonstrate that upregulation of a functional serine synthesis pathway is a metabolic hallmark of B cell activation and the germinal center reaction. Inhibition of phosphoglycerate dehydrogenase (PHGDH), the first and rate-limiting enzyme in this pathway, led to defective germinal formation and impaired high-affinity antibody production. In addition, overexpression of enzymes involved in serine synthesis was a characteristic of germinal center B cell–derived lymphomas, with high levels of expression being predictive of reduced overall survival in diffuse large B cell lymphoma. Inhibition of PHGDH induced apoptosis in lymphoma cells, reducing disease progression. These findings establish PHGDH as a critical player in humoral immunity and a clinically relevant target in lymphoma.

## Graphical abstract

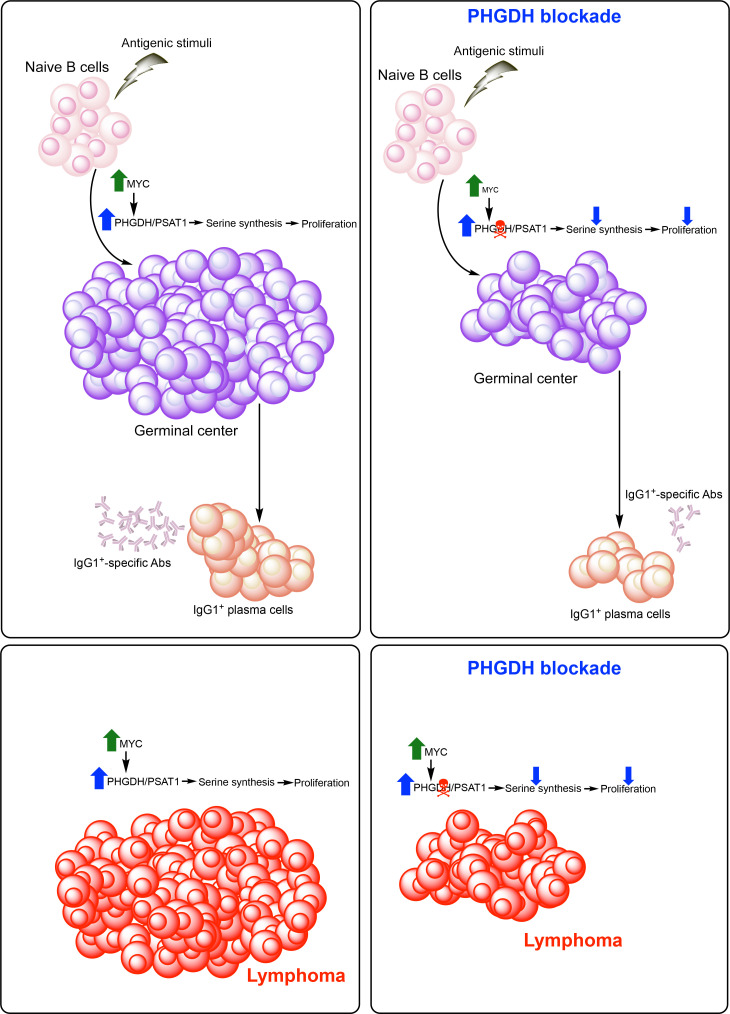

## Introduction

B cells play a critical role in the humoral immune response that eliminates threats to the host by secreting highly specific antibodies. Activated B cells (ABCs) can either differentiate into extrafollicular plasmablasts essential for early protective immune responses or enter a germinal center (GC). In the GC, B cells undergo affinity maturation and eventually differentiate into plasma cells, which secrete high-affinity antibody critical to eliminate the infectious agent, or memory B cells that confer long-lasting protection from secondary infection (1–4). In the GC reaction, B cells are activated by antigen engagement via their B cell receptor (BCR) and subsequent CD4^+^ T cell help, leading to MYC induction (5). The cell-cycle regulator *MYC* is essential for the formation and maintenance of GCs (6). *MYC* is commonly dysregulated in many high-grade B cell malignancies, including GC-derived lymphomas such as Burkitt lymphoma (BL) and diffuse large B cell lymphoma (DLBCL; ref. 7). *MYC* is a master regulator of metabolism, regulating the activity of many metabolic pathways including glycolysis and glutaminolysis. B cell proliferation, either in the context of a GC reaction or in B cell lymphomas, requires significant alterations in cellular metabolism to sustain the demands of dividing cells (8–10). B cells upregulate glycolysis following BCR engagement, a metabolic switch that is also characteristic of many cancers, including high-grade lymphomas (11–17). However, little is known about which metabolic pathways are involved in the utilization of glucose to support proliferating B cells.

One pathway that has emerged as a key metabolic node in cellular proliferation is the serine synthesis pathway (SSP). This uses a downstream product of glycolysis, 3-phosphoglycerate, to produce serine by the action of phosphoglycerate dehydrogenase (PHGDH), phosphoserine aminotransferase 1 (PSAT1), and phosphoserine phosphatase (PSPH) (18). Notably, all 3 of these enzymes are known MYC targets (19). Serine is necessary for glycine synthesis and phospholipid production, and it acts as a 1-carbon donor to the folate cycle, with serine-derived 1-carbon units being used for the synthesis of purine nucleotides to support cell growth (18, 20–22). Overexpression of SSP enzymes and increased serine biosynthesis from glucose is a feature of many types of cancer (23). While some cancers acquire amplification or overexpression of *PHGDH*, the first and rate-limiting step in this pathway, other types of cancers activate oncogenes such as *MYC*, *MDM2*, *KRAS*, and *NRF2*, leading to increased SSP enzyme expression (19, 23–26). Upregulation of the SSP allows cells to increase de novo synthesis of serine from glucose when extracellular serine availability is limiting in the tumor microenvironment (27–33). In support of this, a recent study has shown that inhibition of PHGDH is able to attenuate the growth of brain metastasis in vivo (34–36). Despite this, little is known about the role of the SSP in B cell lymphoma, with no reports on its role in normal GC biology. Here, we performed a comprehensive characterization of the role of SSP in the GC reaction and lymphomagenesis. We reveal that upregulation of the SSP is a metabolic hallmark of B cell activation and lymphoma, with PHGDH being a critical player in humoral immunity and a clinically relevant target in lymphoma.

## Results

### Resting human naive B cells lack expression of SSP enzymes that are induced upon activation.

**Figure 1:**
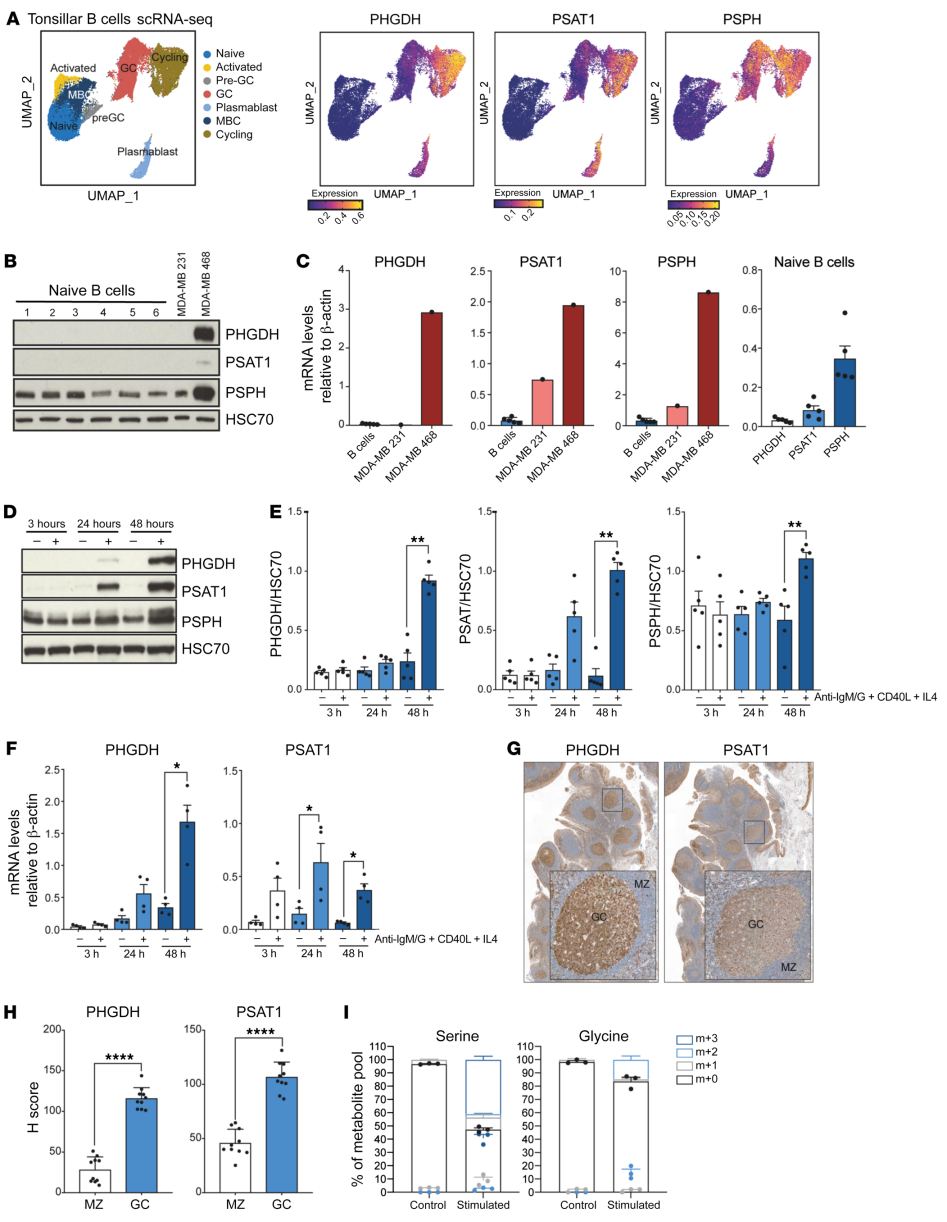
Upregulation of the SSP is a metabolic hallmark of GC B cells. (**A**) Uniform Manifold Approximation and Projection (UMAP) of tonsillar B cell single-cell RNA clusters (including naive, activated, pre-GC, total GC, plasmablasts, memory [MBC], and cycling B cells) (left). Expression of SSP-network genes in B cell subsets (right). (**B**) Analysis of PHGDH, PSAT1 and PSPH protein levels in human naive B cells isolated from blood bank volunteers by immunoblotting (*n =* 6). MDA-MB-231 and MDA-MB-468 cell lines were used as control for low and high SSP-enzyme expression, respectively. (**C**) Quantification of specific transcript levels relative to β-actin mRNA levels. (**D**) Representative immunoblot of PHGDH, PSAT1, and PSPH in resting and activated human naive B cells. Human B cells were left unstimulated (–) or stimulated (+) with anti-IgM/G antibody, CD40 ligand (CD40L), and IL-4 for 3, 24, and 48 hours. (**E**) Quantification of protein levels shown in **D** normalized to HSC70. (**F**) Relative mRNA expression of SSP enzyme genes in resting and activated human B cells determined by qPCR. Isolated human B cells were left unstimulated (–) or stimulated with (+) with anti-IgM/G antibody, CD40L, and IL-4 for 3, 24, and 48 hours before mRNA extraction. Transcript levels were determined relative to β-actin mRNA levels (*n =* 4). (**G** and **H**) Representative IHC staining for PHGDH and PSAT1 in germinal center (GC) and mantle zone (MZ) areas in sequential sections of human reactive tonsils (×5 and ×20 magnification) and quantification (*n =* 10). (**I**) Mass isotopologue distribution of U-[^13^C]-glucose–derived serine and glycine from human resting and activated B cells. B cells were unstimulatedor stimulated with anti-IgM/G antibody, CD40L, and IL-4 for 48 hours. Cells were cultured for 2 hours in serine/glycine deplete media containing U-[^13^C]-glucose. Data are shown as the mean ± SEM. *P < 0.05, **P < 0.01, and ****P < 0.0001, by Mann-Whitney *U* test (**E**, **F**, and **H**).

To understand the role of the SSP during B cell responses in vivo, we analyzed the expression of SSP genes in B cell subsets isolated from reactive human tonsils of cancer-free individuals by single-cell RNA-Seq (37). We observed elevated expression of *PHGDH*, *PSAT1*, and *PSPH* in cycling GC B cells compared with naive and nonproliferating B cells, suggesting an important role of the SSP in B cell proliferation (Figure 1A). We then examined the expression of SSP enzyme proteins and mRNA in naive B cells isolated from the peripheral blood of healthy individuals (Supplemental Figure 1; supplemental material available online with this article; https://doi.org/10.1172/JCI153436DS1). While resting naive B cells expressed very low to negligible amounts of PHGDH and PSAT protein, these enzymes were robustly induced 24–48 hours after stimulation by anti-IgM/G, CD40L, and IL-4 — signals that mimic those delivered in vivo to induce GC responses and B cell proliferation (Figure 1, B–E). In contrast, PSPH was constitutively expressed, becoming further elevated after stimulation (Figure 1, B–E). Treatment of naive B cells by these stimuli alone or in combination revealed that upregulation of PHGDH and PSAT was predominantly driven by BCR stimulation, which was synergistic with costimulation by CD40L and/or IL-4 (Supplemental Figure 2, A and B). Treatment of naive B cells by CpG to activate B cells via TLRs also resulted in induction of PHGDH and PSAT1 expression, although not to the degree of that seen after stimulation by the combination of anti-IgM/G, CD40L, and IL-4 (Supplemental Figure 2C). The temporal dynamics of PHGDH and PSAT1 were noted to be different, with PSAT1 expression being induced more rapidly than PHGDH, a pattern also observed in their mRNA transcripts (Figure 1, E and F). Importantly, IHC analysis of reactive human tonsils showed striking expression of PHGDH and PSAT1 within GCs, but not in mantle zone (MZ) areas (Figure 1, G and H, and Supplemental Figure 2D), confirming the upregulation of these enzymes as a hallmark of human GC B cells in vivo. We next assessed the dynamics of serine metabolism in ABCs. We cultured isolated human B cells with U-[^13^C]-glucose and examined the steady-state incorporation of ^13^C-glucose–derived carbon into serine and glycine using liquid chromatography–mass spectrometry (LC-MS). While resting B cells fail to incorporate U-[^13^C]-glucose into serine, approximatively 50% of the intracellular serine pool was labeled from glucose in stimulated B cells, with 40% of serine carbon being fully labeled (Figure 1I). When serine is directly derived from fully labeled glucose, it can be expected that all 3 of serine’s carbons will carry the ^13^C label (m+3). However, partially labeled serine isotopologues (m+1 and m+2) were also detected, likely due the interconversion of ^13^C-labeled and unlabeled serine and glycine (Figure 1I), indicating the bidirectional nature of this pathway. Taken together, these data indicate that resting human naive B cells lack expression of SSP enzymes, which are induced upon activation to provide a functional ability to synthesize serine and glycine from glucose.

### Characterization of the SSP in mice after activation in vivo.

**Figure 2:**
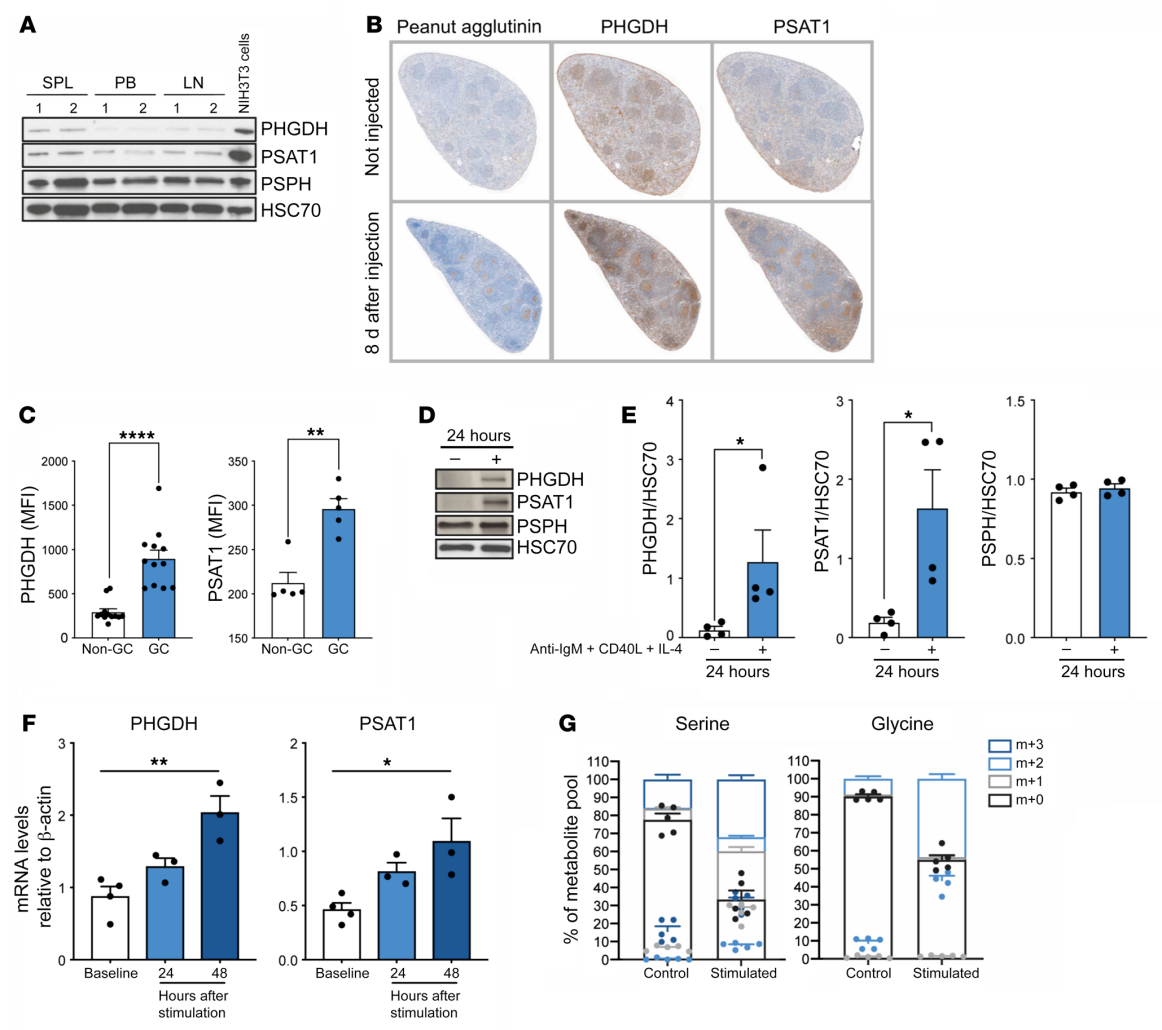
Characterization of the SSP in WT mice after activation in vivo. (**A**) Analysis of PHGDH, PSAT1, and PSPH protein levels in resting B cells isolated from mouse spleen (SPL), peripheral blood (PB), and lymph nodes (LN). NIH3T3 murine cells were used as control for high expression of SSP-related enzymes. (**B**) Representative IHC staining for PNA as GC marker, PHGDH, and PSAT1 on consecutive spleen sections derived from mouse spleens 8 days after sheep RBC immunization (×5 magnification). (**C**) Expression of PHGDH and PSAT1 in GC B cells and non-GC B cells harvested from mouse spleen 8 days after immunization with sheep RBC. (**D**) Representative immunoblots of PHGDH, PSAT1, and PSPH proteins levels in murine resting and activated B cells. (**E**) Mouse B cells were isolated from spleen and left unstimulated (–) or stimulated (+) with anti-IgM/G antibody, CD40L, and IL-4 for 24 hours before protein extraction and quantification of protein levels normalized to HSC70. Individual samples (dots) and means (bars) values are plotted (*n =* 4). (**F**) Relative mRNA expression of SSP enzyme genes in resting and activated mouse B cells as determined by qPCR. Isolated mouse B cells were left unstimulated or stimulated with anti-IgM/G antibody, CD40L, and IL-4 for 24 and 48 hours before mRNA extraction. Specific transcript levels were determined relative to β-actin mRNA levels (*n =* 4). (**G**) Mass isotopologue distribution of U-[^13^C_6_]-glucose–derived serine and glycine from murine resting and activated murine B cells. Cells were left unstimulated or stimulated with anti-IgM/G antibody, CD40L, and IL-4 for 48 hours. Cells were then cultured for 2 hours in serine/glycine-deplete media containing U-[^13^C]-glucose. ^13^C isotopologue distribution in serine and glycine was determined by LC-MS. Data are shown as the mean ± SEM. *P < 0.05, **P < 0.01, and ****P < 0.0001, by Mann-Whitney *U* test (**C**, **E**) or by 1-way ANOVA (**F**).

We characterized the expression of SSP enzymes in healthy resting murine B cells isolated from different tissue compartments (Supplemental Figure 3A). Consistent with the human data, there was low expression of PHGDH and PSAT1 protein in the peripheral blood, spleen, and lymph nodes of WT mice (Figure 2A). IHC analysis of murine spleen and lymph nodes also confirmed low expression of SSP enzymes in resting B cells (Figure 2B). The expression of PHGDH and PSAT1 was assessed in murine spleen and lymph nodes by flow cytometry and IHC 8 days after immunization with sheep RBCs, a T cell–dependent antigen that elicits robust GC responses. PHGDH and PSAT1 were specifically detected within peanut agglutinin^+^ (PNA^+^) GCs in the spleen (Figure 2, B and C, and Supplemental Figure 3B) and lymph nodes (Supplemental Figure 3C). Expression of SSP-involved enzymes was also increased in splenic B cells following in vitro stimulation for 24–48 hours (Figure 2, D–F, and Supplemental Figure 3D). Comparable with human B cells, activation of murine B cells resulted in a clear increase in their ability to synthesize serine and glycine from U-[^13^C]-glucose (Figure 2G).

### Genetic loss and pharmacological inhibition of PHGDH impairs GC responses.

**Figure 3:**
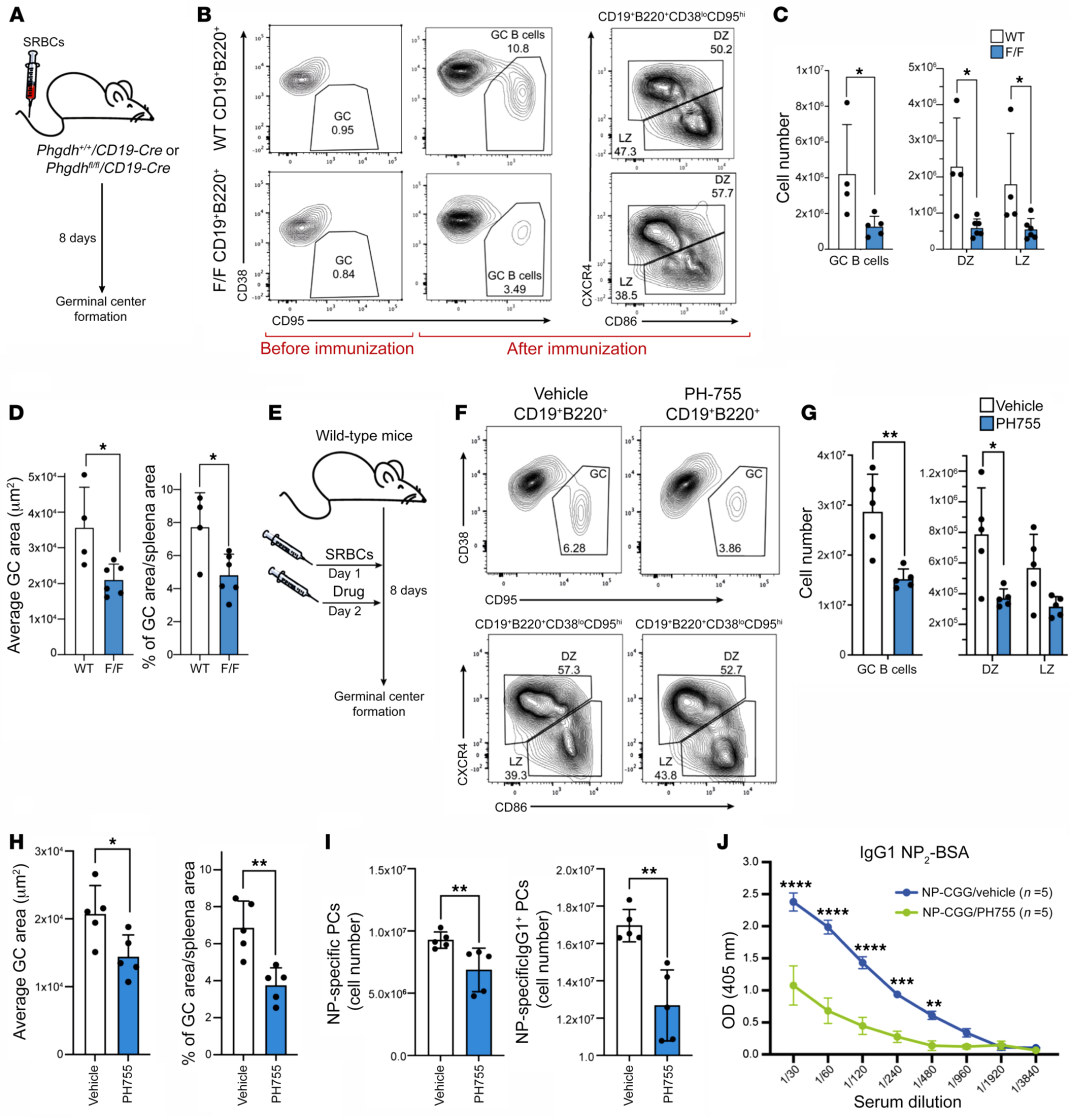
Genetic loss and pharmacological inhibition of PHGDH impairs GC responses. (**A**) Mice were injected with sheep RBCs and spleens examined by IHC and flow cytometry 8 days after immunization. (**B**) Representative flow cytometric analysis of splenic B cells from *Phgdh^+/+^;Cd19-Cre* (WT) or *Phgdh^fl/fl^;Cd19-Cre* (F/F) before and after immunization to identify GC B cells (CD19^+^B220^+^CD38^lo^CD95^hi^), as well as DZ (CD86^lo^CXCR4^hi^) and LZ (CD86^hi^CXCR4^lo^) B cells, within GC splenic population. (**C**) Flow cytometric analysis of absolute numbers of B cell subsets within CD19^+^B220^+^CD38^lo^CD95^hi^ splenic population from *Phgdh^fl/fl^;Cd19-Cre* (*n =* 6) and *Phgdh^+/+^;Cd19-Cre* (*n =* 4) after immunization. (**D**) Average GC area (left) and proportion (%) of GC area per spleen area (right) from *Phgdh^fl/fl^;Cd19-Cre* (F/F) and *Phgdh^+/+^;Cd19-Cre* (WT) mice after immunization. (**E**) WT mice were immunized with sheep RBCs 1 day before PH-755 treatment (300 mg/kg PH-755 orally twice daily for 7 days). Spleens were analyzed 8 days after immunization. (**F**) Representative flow cytometric analysis of splenic B cells from mice immunized with sheep RBCs and treated with vehicle/PH-755 to identify GC, DZ, and LZ B cells within GC splenic population. (**G**) Flow cytometric analysis of absolute number of GC, DZ, and LZ B cells within B220^+^ splenocytes collected from mice 8 days after sheep RBCs immunization; mice were treated with vehicle (*n =* 5) or PH-755 (*n =* 5). (**H**) Average GCs area (left) and proportion (%) of GC area per spleen area (right) from vehicle- and PH-755–treated mice 8 days after sheep RBC immunization. (**I**) Summary of NP_2_-specific plasma cells (PCs; left) and NP_2_-specific IgG1 PCs (right; total number per popliteal lymph nodes). WT mice were treated with either vehicle (*n =* 5) or PH-755 (*n =* 5) for 7 days. Animals were injected with NP-CGG 1 day before PH-755 treatment. Popliteal lymph nodes were collected 8 days after NP-CGG immunization. (**J**) Serum antibody titers for NP_2_-specific IgG1 8 days after NP-CGG immunization. Data are shown as the mean ± SEM. *P < 0.05, **P < 0.01, ***P < 0.001, and ****P < 0.0001, by Mann-Whitney *U* test (**C**, **G**, **I**, and **J**) or unpaired *t* test (**D** and **H**).

To interrogate the role of the SSP in B cell differentiation and GC responses, we targeted PHGDH, the first enzyme in the SSP, genetically and pharmacologically. We generated a conditional KO murine model in which *Phgdh* was specifically deleted in B cells by crossing mice carrying floxed *Phgdh* alleles with mice expressing the Cre recombinase under control of the *Cd19* promoter (*Phgdh^fl/fl^;Cd19-Cre*). As expected, B220^+^ B cells isolated from these mice did not show an increase in PHGDH expression following in vitro stimulation with anti-IgM/G antibody, CD40L, and IL-4, but they retained the ability to upregulate PSAT1 (Supplemental Figure 4A). We then analyzed animals before and 8 days after immunization with sheep RBCs to assess the impact of deletion of *Phgdh* on GC responses (Figure 3A). Numbers of B220^+^CD38^+^Fas^+^ GC B cells were reduced in *Phgdh^fl/fl^;Cd19-Cre* mice following immunization, reflecting a reduction in both light zone (LZ) and dark zone (DZ) B cells (Figure 3, B and C). These observations were also confirmed by IHC analyses showing a significant reduction in average PNA^+^ GC area and proportion of splenic sections occupied by PNA^+^ GCs in *Phgdh^fl/fl^;Cd19-Cre* mice (Figure 3D and Supplemental Figure 4B). Further confirmation of the B cell–specific nature of the *Phgdh* KO was provided by a complete absence of PHGDH expression in GCs, in contrast to PSAT1, while PHGDH was expressed in the T cell–rich periarteriolar lymphoid sheaths (Supplemental Figure 4C). Thus, conditional KO of *Phgdh* in B cells results in an impaired GC response. Notably, we did not observe any significant difference in numbers of Pro-B cells, Pre-B cells, immature B cells, and mature B cells in BM when comparing *Phgdh^fl/fl^;Cd19-Cre* and *Phgdh^+/+^;Cd19-Cre* mice (Supplemental Figure 5, A and B). In addition, there was no significant difference in numbers of transitional, marginal zone, and follicular B cells in the spleen when comparing *Phgdh^fl/fl^;Cd19-Cre* and *Phgdh^+/+^;Cd19-Cre* mice (Supplemental Figure 5, C–F). We would hypothesize that these observations can be explained by lower deletion efficiency of Cd19-Cre in BM B cells (38), combined with the relative impact of *Phgdh* deletion on B cells proliferating in serine-deplete microenvironments.

We next assessed whether inhibition of GC responses could be replicated by pharmacological inhibition of PHGDH using a specific inhibitor, PH-755 (35, 36). Mice were injected with sheep RBCs 24 hours before starting treatment with PH-755 or vehicle control. GC responses were assessed by flow cytometry and IHC as before (Figure 3E). Treatment with PH-755 resulted in significantly reduced numbers of GC B cells comparable with that seen with the conditional KO (Figure 3, F and G). IHC analysis also showed a reduction in PNA^+^ GC area, with an overall reduction in the proportion of splenic sections occupied by PNA^+^ GC B cells but with preservation of PHGDH expression (Figure 3H and Supplemental Figure 6, A and B). We then proceeded to assess the impact on NP-specific plasma cell responses after 4-hydroxy-3-nitrophenyl acetyl–chicken γ-globulin (NP-CGG) immunization. PH-755 treatment resulted in a significant reduction of numbers of NP-specific GC B cells and total NP-specific and NP-specific IgG1 plasma cells that correlated with reduced titres of anti–NP IgG1 antibodies in the sera of these mice (Figure 3, I and J), with no effect on NP-specific IgM responses (Supplemental Figure 6, C–E). The lack of impact of PHGDH inhibition on NP-specific IgM responses could, therefore, reflect increased extracellular serine availability outside of GCs, leading to a relative sparing of extrafollicular responses. Taken together, these data show that PHGDH inhibition impairs GC formation with a resultant reduction in high-affinity antibody production.

### PHGDH inhibition impairs B cell proliferation and de novo serine and glycine synthesis.

**Figure 4:**
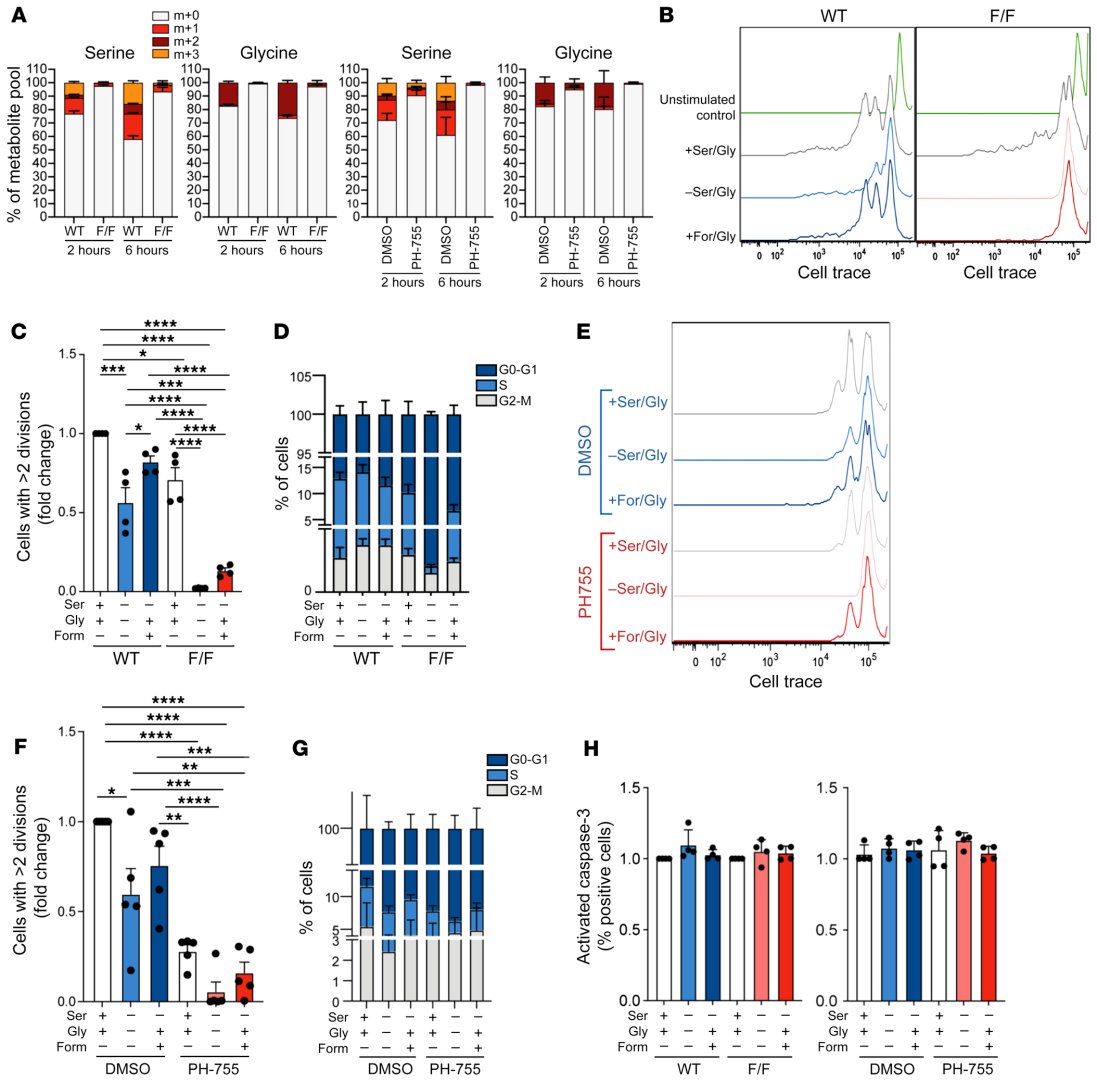
PHGDH inhibition impairs B cell proliferation and de novo serine and glycine synthesis. (**A**) Mass isotopologue distribution of U-[^13^C_6_]-glucose–derived serine and glycine in B220^+^ B cells isolated from *Phgdh^+/+^;Cd19-Cre* (WT) or *Phgdh^fl/fl^;Cd19-Cre* (F/F) or WT C57BL/6J mice. Isolated cells were stimulated with anti-IgM/G antibody, CD40L, and IL-4 for 48 hours and were then cultured for 2 hours in serine/glycine-deplete media with U-[^13^C]-glucose (and treated with/without PH-755 for WT B cells). (**B** and **C**) Representative proliferation profiles and quantification of B220^+^ B cells from either *Phgdh^+/+^;Cd19-Cre* (WT) or *Phgdh^fl/fl^;Cd19-Cre* (F/F) mice. Cells were labeled with the Cell Proliferation Dye eFluor 670 and then cultured for 3 days with anti-IgM/G antibody, CD40L, and IL-4 in complete media, serine/glycine-free medium, or serine/glycine-free medium containing glycine and formate (*n =* 4 per genotype). (**D**) Cell-cycle analysis of B220^+^ cells isolated from *Phgdh^+/+^;Cd19-Cre* (WT) or *Phgdh^fl/fl^;Cd19-Cre* (F/F) mice. Cells were cultured for 48 hours with anti-IgM/G antibody, CD40L, and IL-4 in complete media, serine/glycine-free medium, or serine/glycine-free medium containing glycine and formate; they then underwent BrdU labeling and 7-AAD staining to assess cell cycle. (**E** and **F**) Representative proliferation profiles and quantification of B220^+^ B cells from WT mice. Cells were labeled with the Cell Proliferation Dye eFluor 670 and were then cultured for 3 days with anti-IgM/G antibody, CD40L, and IL-4 in complete media, serine/glycine-free medium, or serine/glycine-free medium containing glycine and formate in combination with DMSO or 10 μM PH-755 (*n =* 5 per group). (**G**) Cell-cycle analysis of B220^+^ B from WT mice. Cells were cultured for 48 hours with anti-IgM/G, CD40L, and IL-4 in complete medium, serine/glycine-free medium, or serine/glycine-free medium containing glycine and formate in combination with DMSO or 10 μM PH-755 before cell-cycle analysis. (**H**) Activate Caspase-3 on B220^+^ B cells from either *Phgdh^+/+^;Cd19-Cre* (WT) or *Phgdh^fl/fl^;Cd19-Cre* (left panel; *n =* 4 per group) and treated as described in **D**, and on B cells isolated from WT mice (right panel; *n =* 3 per group) and treated as described in **G**. Data are shown as the mean ± SEM. *P < 0.05, **P < 0.01, ***P < 0.001 and ****P < 0.0001, by 1-way ANOVA (**C** and **F**).

Based on previous observations showing the crucial role of the SSP in cell growth and proliferation, we proceeded to assess the impact of PHGDH inhibition on the behavior of primary stimulated murine B cells. Since GC B cells cannot survive ex vivo due to the rapid inception of a proapoptotic program (39), we performed these experiments on B220^+^ cells isolated from spleens. Stimulated primary B cells from either *Phgdh^fl/fl^;Cd19-Cre* mice or *Phgdh^+/+^;Cd19-Cre* mice were tested for their ability to synthesize serine and glycine from glucose by incubating them with U-[^13^C]-glucose. Comparable experiments were performed with stimulated B cells taken from WT mice treated with either PH-755 or control. As expected, both genetic KO and pharmacological inhibition of PHGDH almost completely abolished the ability of the cells to incorporate labeled glucose into serine at these time points (Figure 4A). We then assessed the impact of PHGDH inhibition on the proliferative capacity of B cells from *Phgdh^fl/fl^;Cd19-Cre* mice in vitro following stimulation, compared with *Phgdh^+/+^;Cd19-Cre* control B cells. B cell proliferation measured by dye-dilution assay (Figure 4B) revealed only a slight reduction in proliferation of *Phgdh^fl/fl^;Cd19-Cre* B cells as compared with *Phgdh^+/+^;Cd19-Cre* cells when stimulated in serine-glycine replete medium (Figure 4C). However, in media lacking serine and glycine, the proliferation of *Phgdh^fl/fl^;Cd19-Cre* B cells was completely abrogated, whereas PHGDH induction following stimulation sustained the proliferation of *Phgdh^+/+^;Cd19-Cre* B cells (Figure 4, B and C). Notably, the proliferative capacity of these PHGDH-deficient B cells could be partially rescued by the addition of glycine with formate as a 1-carbon donor (32). In addition, providing *Phgdh^+/+^;Cd19-Cre* B cells with formate and glycine allowed them to proliferate optimally in the absence of serine (Figure 4, B and C). We then proceeded to investigate whether the changes in proliferation were accompanied by altered cell cycling and/or apoptosis. Notably, the reduction in proliferation was mirrored by a decrease in the fraction of cells in S phase, particularly when genetic KO of *Phgdh* was accompanied by the removal of extracellular serine and glycine (Figure 4D). This decrease in cells in S phase was accompanied by an accumulation of cells in G0/G1 phase (Figure 4D). Supplementing serine-starved *Phgdh^fl/fl^;Cd19-Cre* B cells with glycine and formate partially rescued the fraction of cells in S phase, as reflected by cell proliferation (Figure 4D). We then proceeded to repeat these experiments with WT B cells cultured in various combinations of serine, glycine, and formate, with PH-755 or vehicle control. Treatment with PH-755 largely recapitulated the pattern observed with the genetic ablation of *Phgdh*, with the exception that PH-755 treatment was able to partially inhibit B cell proliferation even in the presence of extracellular serine and glycine (Figure 4, E–G). Notably, we did not observe increased Caspase-3 activation as a marker of apoptosis in the *Phgdh-*KO or drug-treated B cells under any of the conditions (Figure 4H). In summary, these results show that inhibition of PHGDH effectively blocks de novo synthesis of serine and glycine from glucose, which in turn has a cytostatic effect on primary murine B cells in the absence of extracellular serine and glycine.

### Activation of the SSP pathway in human MYC-driven GC lymphomas.

**Figure 5:**
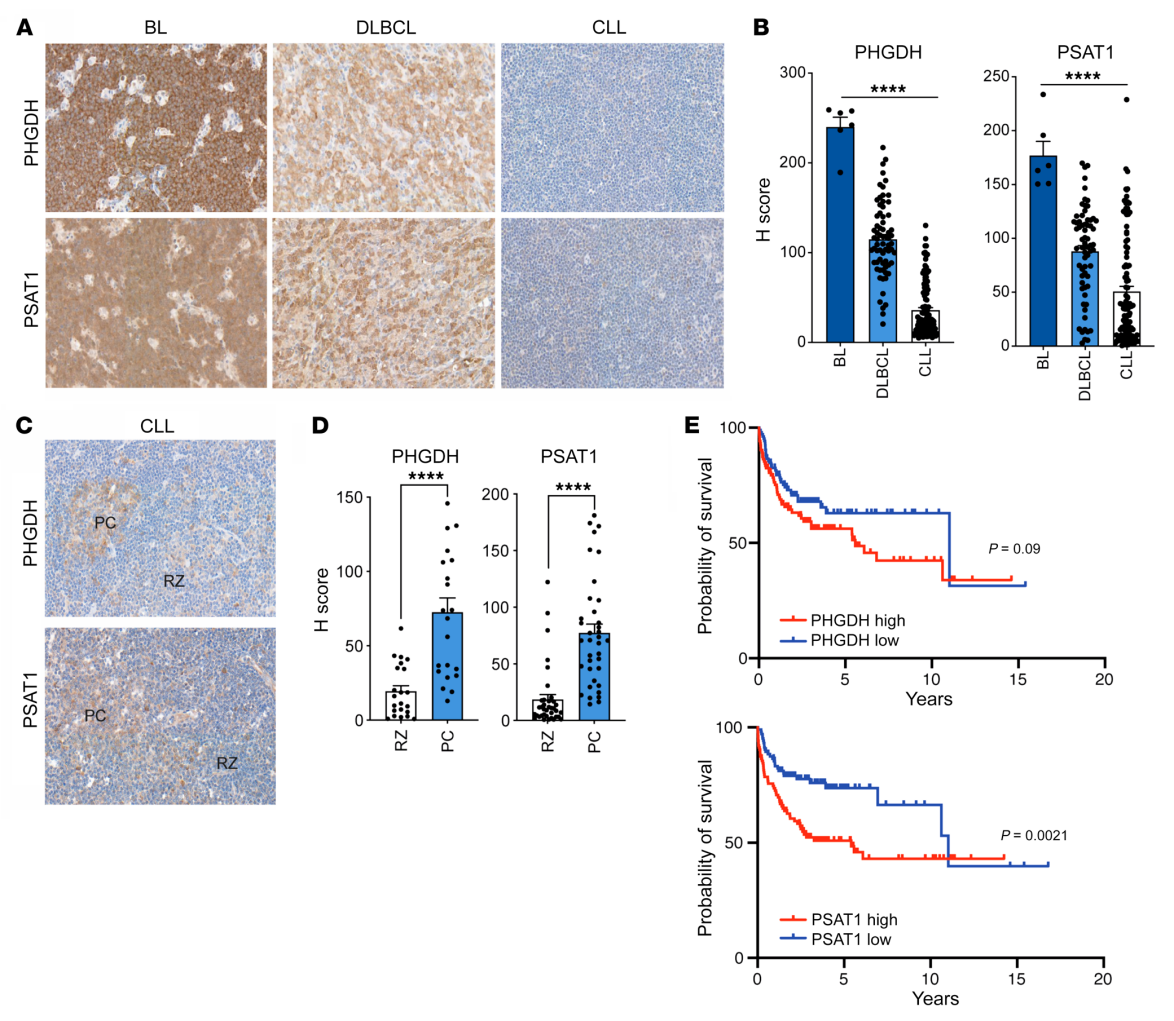
Human GC lymphomas are characterized by activation of the SSP pathway. (**A** and **B**) Representative immunohistochemical staining (×20 magnification) and quantification for PHGDH and PSAT1 abundance in sections of human diagnostic biopsies from patients with Burkitt lymphoma (BL), Diffuse Large B cell lymphoma (DLBCL), and Chronic lymphocytic leukemia (CLL). The statistical difference was analyzed using the ordinary 1-way ANOVA. (**C** and **D**) IHC analysis (×40 magnification) for PHGDH and PSAT1 in proliferation centers (PC) and resting zone (RZ) areas in sections from biopsies collected from patients with chronic lymphocytic leukemia (CLL), and quantification. Individual samples (dots) and means (bars) values are plotted. The statistical difference was analyzed using the Mann-Whitney *U* test. (**E**) Kaplan-Meier survival analysis of patients with DLBCL from a published data set (GSE10846) (44). Patients whose *PHGDH/PSAT1* mRNA levels were within the top quartile were grouped as *PHGDH/PSAT1* high; those with *PHGDH/PSAT1* mRNA levels within the bottom quartile were grouped as *PHGDH/PSAT1* low. Data are shown as the mean ± SEM. ****P < 0.0001, by 1-way ANOVA (**B**) or by Mann-Whitney *U* test (**D**). Survival analysis were conducted with log-rank (Mantel-Cox) test (**E**).

One of the key emerging concepts in the cancer metabolism field over the last few years is that the metabolic phenotype of cancer cells reflects their cell-of-origin in combination with other factors such as oncogenic drivers and the tumor metabolic microenvironment (40). In light of this, we analyzed the role of the SSP in GC lymphomas. These were of particular interest due to the role of MYC in their pathogenesis and as a regulator of the SSP. The expression of PHGDH and PSAT1 was assessed by IHC in a series of diagnostic biopsies from patients with BL, DLBCL, and chronic lymphocytic leukemia (CLL). Notably, very high expression of these 2 proteins was observed in BL, consistent with a recent report (41), with intermediate to high expression in DLBCL and relatively low expression in CLL (Figure 5, A and B). Although biopsies from CLL patients showed the lowest expression, PHGDH and PSAT1 staining was significantly increased within proliferation centers, which are microanatomical sites in lymphoid tissues where CLL cells proliferate and where MYC is expressed (Figure 5, C and D; refs. 42, 43). Given the heterogeneity of expression in DLBCL patients, we next interrogated a published data set (GSE10846; https://www.ncbi.nlm.nih.gov/) to investigate the relationship between SSP gene expression and patient survival (44). Importantly, high expression of *PSAT1* was significantly associated with poorer overall survival in DLBCL (Figure 5E), with a trend toward patients with high expression of *PHGDH* also having reduced overall survival. The expression of *PHGDH* and *PSAT1* in ABC-like or GC B cell–like (GCB-like) DLBCL was also assessed due to the prognostic importance of these profiles (45). There was no difference between the expression of *PHGDH* and *PSAT1* between these 2 subsets, although there was a weak positive correlation between *MYC* expression and the expression of SSP enzymes (Supplemental Figure 7, A and B). The lack of difference in *PHGDH* and *PSAT1* expression when comparing ABC- and GCB-like DLBCL likely reflects our observations regarding the strong induction of these 2 enzymes upon activation of human B cells both in vitro and in GCs in vivo (Figure 1, D–H). Overall, the upregulation of the SSP is a feature of GC malignancies and can predict impaired overall survival in DLBCL.

### PHGDH inhibition impairs proliferation and promotes apoptosis in BL cells.

**Figure 6:**
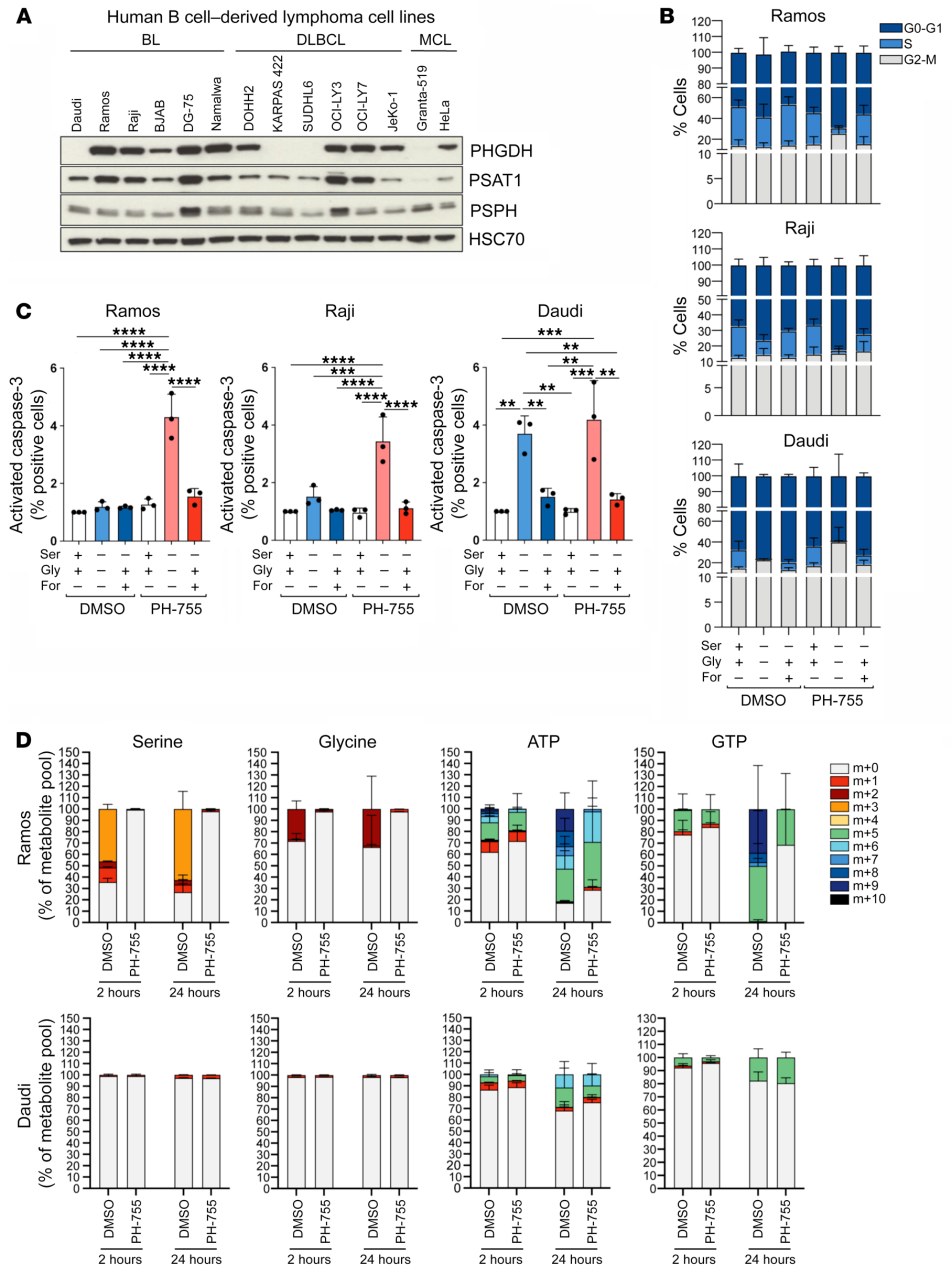
PHGDH inhibition impairs proliferation and promotes apoptosis in Burkitt lymphoma cells. (**A**) Western blot analysis of PHGDH, PSAT1, and PSPH protein expression in B cell–derived lymphoma cell lines (mantle cell lymphoma [MCL]). Representative of 3 independent experiments. HSC70 was used as loading control. (**B**) Cell cycle profile of Ramos (top), Raji (center), and Daudi (bottom) cells. Cells were plated either in complete medium or equivalent medium lacking serine and glycine supplemented or not with 0.5 mM sodium formate and 0.4 mM glycine and treated with DMSO (as a solvent control) or 10 μM PH-755, followed by incubation with 10 μM BrdU and by staining with anti-BrdU and 7-ADD. Data are presented as mean ± SEM and are representative of 3 independent experiments, with value for DMSO-treated cells and cultured in complete medium set to 1.0. (**C**) Ramos (left), Raji (center), and Daudi (right) cells were cultured in the same conditions specified in **B** for 48 hours. Cells were then permeabilized, fixed, and stained for active Caspase-3. Positive cells for active Caspase-3 were analyzed by flow cytometry. Graph shows the mean derived from 3 independent experiments, with value for DMSO-treated cells and cultured in complete medium set to 1.0. Data are shown as the mean ± SEM. **P < 0.01, ***P < 0.001, and ****P < 0.0001, by 1-way ANOVA with Tukey’s post hoc test. (**D**) Mass isotopologue distribution of U-[^13^C_6_]-glucose–derived serine and glycine for Ramos (top) and Daudi (bottom) cells cultured for 2 and 24 hours in medium lacking serine and glycine in presence of U-[^13^C_6_]-glucose (10 mM) and treated with DMSO or 10 μM PH-755. Serine, glycine, ATP, and GTP levels were measured by LC-MS. The percentage distribution of each isotopologue for their respective metabolite pool is shown. Data are presented as mean ± SEM of 6 repeats and are representative of 3 independent experiments.

We hypothesized that the SSP may represent a therapeutic target in human lymphoma. The expression of SSP enzymes was assessed in a panel of human lymphoma cell lines (Figure 6A and Supplemental Figure 8A). In contrast to other BL cell lines, Daudi cells had no expression of PHGDH but did express PSAT1 and PSPH. Consequently, Daudi cells were unable to enter S phase when cultured without extracellular serine and glycine, with cycling being partially rescued by glycine and formate. In contrast, Ramos and Raji cells were able to maintain the cell cycle in the absence of serine and glycine (Figure 6B). However, when Ramos and Raji cells were treated with PH-755 to inhibit PHGDH, they also became unable to cycle in the absence of serine and glycine, with PHGDH inhibition having no further effect on Daudi cells. In contrast to primary B cells, we observed significantly increased Caspase-3 activation in Daudi cells cultured in serine/glycine-deplete conditions and in all 3 cell lines when serine-glycine deprivation was combined with PHGDH inhibition with PH-755 (Figure 6C).

We assessed the ability of these cell lines to synthesize serine, glycine, and the purine nucleotides adenosine triphosphate (ATP) and guanine triphosphate (GTP) from glucose. The cell lines were cultured with U-[^13^C]-glucose for 2–24 hours in the absence of serine and glycine, with labeling being assessed by LC-MS. Ramos and Raji cells grown in the absence of serine and glycine diverted glucose into de novo serine and glycine synthesis, which was prevented when treated with PH-755 (Figure 6D and Supplemental Figure 8B). Furthermore, labeling of higher isotopologues (≥m+6) of ATP and GTP demonstrated that these nucleotides were being synthesized via the SSP, and this could again be inhibited by PH-755 (Figure 6D and Supplemental Figure 8B). Unlike Ramos and Raji cells, this labeling was virtually absent in Daudi cells, consistent with their lower ability to proliferate in the absence of exogenous serine (Figure 6D). In conclusion, these results show that inhibition of PHGDH in the absence of extracellular serine and glycine is able to inhibit purine synthesis and block entry into the S phase of the cell cycle and promote apoptosis of human lymphoma cell lines.

### Genetic loss and pharmacological inhibition of PHGDH reduces lymphoma progression in vivo.

**Figure 7:**
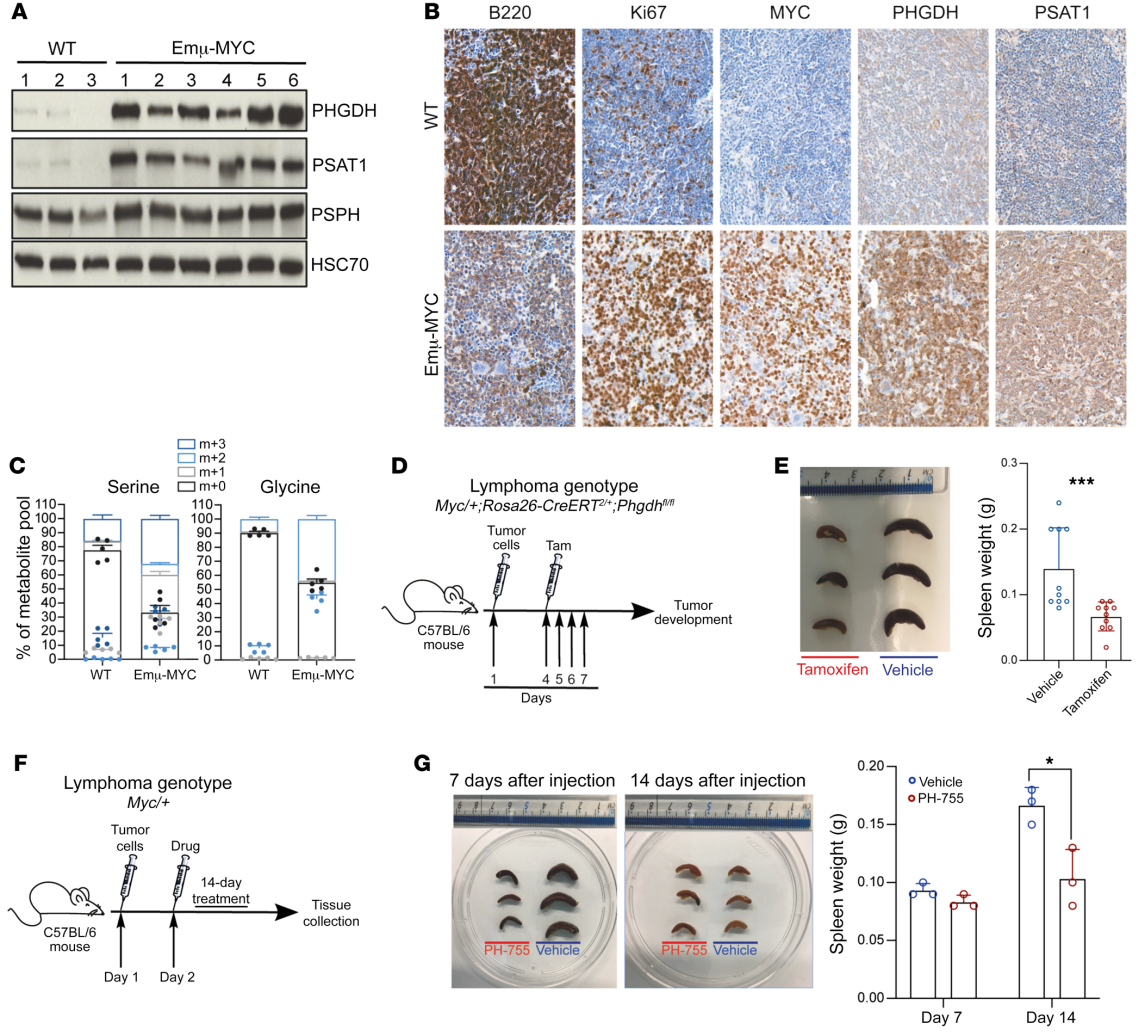
Genetic loss and pharmacological inhibition of PHGDH reduces lymphoma progression in vivo. (**A**) Immunoblot of PHGDH, PSAT1, and PSPH expression in splenic B cells from WT (*n =* 3) and *Eμ-Myc* mice (*n =* 6). HSC70 was used as loading control. (**B**) Representative IHC staining (×20 magnification) for B220, Ki67, MYC, PHGDH, and PSAT1 abundance in sections of spleens from either WT (*n =* 3) or *Eμ-Myc* (*n =* 3) mice. (**C**) Isotope tracing analysis in splenic B cells isolated from either C57BL/6J WT mice or Eμ-MYC mice and cultured for 2 hours with ^13^C_6_-labeled glucose. Serine and glycine levels were measured by LC-MS. The percent distribution of each isotopologue of their respective metabolite pool is represented as mean ± SEM of triplicate cultures and is representative of 3 independent experiments. (**D**) Schematic showing lymphoma transplantation model, in which *Myc/+;Rosa26-CreER^T2/+^;Phgdh^fl/fl^* lymphoma cells are injected via the tail vein into 9-week-old male C57BL/6J mice. Three days after lymphoma engraftment, mice were randomized to receive either vehicle or tamoxifen treatment by oral gavage for 4 days. Samples were collected 20 days after injection. (**E**) Representative pictures of spleens from mice (*n =* 3 per group) sacrificed 20 days after transplantation (left), and quantification of the spleen weight (right). (**F**) Schematic showing lymphoma transplantation model, in which *Myc/+* lymphoma cells are injected via the tail vein into 9-week-old male C57BL/6J mice. Two days after lymphoma engraftment, mice were randomized to be treated with either vehicle or PH-755 by oral gavage for 14 days. (**G**) Representative pictures of spleens from mice (*n =* 3 per group) sacrificed after 7 or 14 days after transplantation (left), and quantification of the spleen weight (right). Data are shown as the mean ± SEM. *P < 0.05 and ***P < 0.001, by 2-tailed Student’s *t* test (**E** and **G**).

To investigate the importance of the SSP pathway in tumor development in vivo, we used the *Eμ-Myc* mouse model, which carries a transgene mimicking the t(8:14) translocation of *MYC* and *IGH* characteristic of human BL (46). We first characterized these mice by assessing the expression levels of SSP-related enzymes in B cells isolated from WT and lymphoma-bearing *Eμ-Myc* mice. Immunoblotting and IHC showed higher expression of PHGDH and PSAT1 in B cells from lymphoma-bearing *Eμ-Myc* heterozygote mice when compared with WT C57BL/6J syngenic mice (Figure 7, A and B). When we investigated the ability of *Eμ-Myc* B cells to synthesize serine and glycine de novo, we found greater incorporation of labeled U-[^13^C]-glucose in these cells compared with B cells isolated from C57BL/6J mouse spleens (Figure 7C). We then investigated the impact of *Phgdh* deletion on *Myc*-driven tumor development in vivo. Deletion of the first enzyme of the SSP pathway, *Phgdh*, does not prevent development, but *Phgdh*-deficient mice are born with severe neurological defects and die soon after birth (47). To overcome these limitations, we first crossed *Eμ-Myc* mice with *Rosa26-CreER^T2/+^*mice (48), which express a tamoxifen-inducible Cre recombinase enzyme. *Eμ-Myc;Rosa26-CreER^T2/+^* animals were then crossed with *Phgdh^fl/fl^* mice to generate *Eμ-Myc/+;Rosa26-CreER^T2/+^;Phgdh^fl/fl^* mice. Following lymphoma onset, lymphoma cells were purified and transplanted by i.v. injection into C57BL/6J syngenic mice. Preliminary control experiments using lymphoma cells from *Myc/+;Rosa26-CreER^T2/+^;Phgdh^+/+^* mice showed no overt evidence of Cre toxicity (Supplemental Figure 9), but they did show different lymphoma genicity, probably reflecting different cooperating mutations that had evolved to counterbalance the proapoptotic activity caused by enforced Myc expression (49). However, mice transplanted with *Eμ-Myc/+;Rosa26-CreER^T2/+^;Phgdh^fl/fl^* lymphoma cells consistently developed splenomegaly and lymphadenopathy from 7 days after injection onward. The recipients were treated either with vehicle or tamoxifen to excise *Phgdh* specifically in the lymphoma cells (Figure 7D). Notably, KO of *Phgdh* induced by tamoxifen treatment resulted in a significant reduction in spleen weight in mice sacrificed 20 days after injection (Figure 7E). We next evaluated the pharmacological activity of the PHGDH inhibitor PH-755 on tumor development. C57BL/6J syngenic mice were injected with single-transgenic *Eμ-Myc* lymphoma cells and then treated with vehicle or PH-755 (300 mg/kg daily, i.p.) for 14 days (Figure 7F). The mice tolerated PH-755 well, with no overt side effects from treatment. As with the genetic KO, pharmacological inhibition of PHGDH with PH-755 also resulted in a significant reduction in lymphoma progression (Figure 7G). Taken together, these data provide evidence that PHGDH is an effective therapeutic target in *MYC*-driven lymphoma.

## Discussion

Our results establish the importance of the SSP in supporting the proliferation of GC B cells. We show that the SSP is upregulated during B cell activation and is a metabolic hallmark of the GC reaction. Inhibition of PHGDH, either genetically or pharmacologically, leads to a defect in GC formation and a reduction in high-affinity antibody production. In addition, overexpression of SSP enzymes is a characteristic of GC-derived lymphomas, with high levels of expression predicting a poorer prognosis in DLBCL. In contrast to healthy ABCs, where PHGDH blockade has a cytostatic effect, inhibition of this enzyme in lymphoma cells induces apoptosis, likely reflecting the impact of MYC overexpression. Importantly, inhibition of PHGDH, either specifically within lymphoma cells using an inducible KO transplantation model or globally using a pharmacological agent, reduces disease progression, highlighting this enzyme as a potentially novel therapeutic target in lymphoma.

These data highlight the interaction between the availability of extracellular serine and the SSP. Collectively, these results suggest that the concentration of serine is rate limiting in GCs, resulting in a requirement for proliferating B cells to synthesize serine from glucose. A recent study has shown that serine is an essential metabolite for effector T cell proliferation (50). Notably, these authors observed that, while effector T cells also upregulate the SSP after activation, they derive the majority of their serine from extracellular sources, with dietary serine availability dictating T cell responses in vivo. Our data suggest an important difference between T cell and B cell biology, due to the anatomical localization of humoral immune responses to the GC. This raises the intriguing possibility that PHGDH inhibition could selectively target the humoral response with relative preservation of T cell responses, with important implications for the treatment of autoimmune disease and lymphoma.

The role that the SSP plays in cancer is complex and an area of active investigation. Much of the focus has been on the role of PHGDH and its potential as a target for therapy. Notably, while PHGDH does seem to be important for the development of some cancers, including metastatic breast cancer, observations in other cancer models have suggested that this enzyme is dispensable for tumor development (23, 27, 51). A previous report suggested that *MYC*-driven lymphomagenesis can occur in the absence of PHGDH. However, those observations were based on the use of λ-*Myc;Phgdh*^+/–^ mice crossed with *Phgdh*^+/–^ mice, in a system that generated lymphoma-prone mice with varying expression of PHGDH (52). It is quite possible that even low levels of PHGDH permit *MYC*-driven lymphomagenesis, which would explain the differences we observed with our systems. Indeed, *MYC*-driven lymphoma may be particularly susceptible to serine deprivation, as previous work has demonstrated that the progression of lymphoma in *Eμ-Myc* mice can be slowed by reducing the availability of extracellular serine and glycine by dietary restriction (25). This interaction between extracellular serine and PHGDH inhibition may open up new therapeutic opportunities, either in terms of combining pharmacological inhibition of PHGDH with a serine/glycine-free diet or in treating lymphoma in serine/glycine-depleted environments such as the CNS (36). Tumor cells may become increasingly dependent on de novo serine synthesis as the disease progresses, as the increasing mass of cancer cells consumes microenvironmental serine.

Our observations support the hypothesis that it is serine and glycine themselves that are important for lymphoma cell proliferation and survival, as these cells retain the ability to cycle and have low rates of apoptosis when PHGDH is inhibited in serine/glycine-replete conditions. Serine appears to be the more important, given our observations that culture with glycine and formate only partially rescues the cycling of normal and malignant B cells. In addition, flux through the SSP is also known to play roles in other metabolic processes such as redox modulation, α-ketoglutarate production, and generation of oncometabolites such as D-2-hydroxyglutarate (53). Taken together, this work provides important evidence that PHGDH is a viable target for the treatment of pathological B cell proliferation, either for the modulation of humoral immunity or in lymphoma.

## Methods

### Animal studies

Mice (3–5 per cage) were allowed access to food and water ad libitum and were kept in a 12-hour day/night cycle starting at 7:00 until 19:00. Rooms were kept at 21°C at 55% humidity. Mice were allowed to acclimatize for at least 1 week prior to experimentation. They were then randomly assigned to experimental groups. B6;129P2-Phgdh<tm2Bsi> mice were purchased from RIKEN BRC (stock no. RBRC02574; ref. 47). *Eμ-Myc/+* transgenic mice (stock no. 002728) and *Rosa26-CreER^T2/+^* mice (stock no. 008463) were purchase from The Jackson Laboratory. *Cd19-Cre–KI* mice were obtained via the MGC foundation (http://www.mgc-foundation.de/). To test the impact of *Phgdh* deletion on GC response, we generated a mouse model in which *Phgdh* could be conditionally deleted in B cells by crossing *Phgdh^fl/fl^* mice with *Cd19-Cre–KI* mice to obtain *Phgdh^fl/fl^;Cd19-Cre–KO* mice. To investigate the impact of *Phgdh* deletion on *Eμ-Myc*–driven tumor development, *Eμ-Myc/+* mice were crossed with *Rosa26-CreER^T2/+^* mice to generate *Eμ-Myc/+;Rosa26-CreER^T2/+^* mice. *Eμ-Myc/+*;*Rosa26-CreER^T2/+^* mice were then crossed either with *Phgdh^fl/fl^* or *Phgdh^+/+^* mice to generate *Eμ-Myc/+;Rosa26-CreER^T2/+^;Phgdh^fl/fl^* or *Eμ-Myc/+;Rosa26-CreER^T2/+^;Phgdh^+/+^* genotypes. Following lymphoma onset, lymphoma cells from these mice were purified and used for the adoptive transfer lymphoma experiments.

For T cell–dependent immunization, either *Phgdh^fl/fl^;Cd19-Cre* or *Phgdh^+/+^;Cd19-Cre* mice (7–12 weeks old) were injected i.v. with 1 × 10^9^ sheep RBCs (TCS Biosciences, SB054). GCs were analyzed at day 8 after immunization. NP was conjugated to CGG at a ratio of NP_18_-CGG. Mice were injected s.c. on the plantar surface of the foot with 20 μg NP_18_-CGG precipitated in alum (Thermo Fisher Scientific). For PHGDH inhibitor experiments, C57BL/6J mice (7–12 weeks old) were i.v. injected with 1 × 10^9^ sheep RBCs. After 24 hours, mice were randomized to receive vehicle (0.5% methylcellulose [MilliporeSigma, H7509], 0.5% Tween-80 [MilliporeSigma, P8192]) or 300 mg/kg PH-755 (Raze Therapeutics) prepared in vehicle twice a day by oral gavage for the duration of the experiment. GCs were analyzed at day 8 after immunization.

For transplantable lymphoma experiments, *Eμ-Myc* mice harboring lymphomas were euthanized according to licence guidelines (project licence reference P319AE968). Spleens were collected immediately on ice, homogenized, and passed through 70 μm cell strainer in complete RPMI 1640 (Thermo Fisher Scientific, 21875091) supplemented with 10% FCS (Thermo Fisher Scientific, 26400044), 2.5% 1M HEPES buffer (MilliporeSigma, H0887), 1% GlutaMAX (Thermo Fisher Scientific, 35050038), 1% penicillin-streptomycin (Thermo Fisher Scientific, 15140122), and 0.1% 0.05M β-mercaptoethanol (MilliporeSigma, M3148). Splenocyte suspensions were washed in PBS and incubated in ACK Lysis Buffer (LONZA, 0000879569) for 5 minutes at room temperature (RT) to lyse residual erythrocytes. Lymphoma cells were washed and filtered through 70 μm cell strainer. Cells were resuspended in complete media, and 2 × 10^6^ cells in 200 μL were injected into C57BL/6J mice (7–12 weeks old). To investigate the effect of PHGDH inhibition on tumor development, mice were randomized to receive vehicle or 300 mg/kg PH-755 one day after lymphoma injection. Vehicle or drug was administrated once a day by oral gavage for 14 days. Mice were monitored from the conclusion of the treatment to the duration of the experiment. Moribund mice were euthanized according to license guidelines, and lymph nodes and spleens were collected for further analysis.

For PHGDH excision experiments, 2 × 10^6^ *Eμ-Myc/+;Rosa26-CreER^T2/+^;Phgdh^fl/fl^* or *Eμ-Myc/+;Rosa26-CreER^T2/+^;Phgdh^+/+^* cells were injected via tail vein into 7- to 12-week-old C57BL/6J mice. Three days after lymphoma injection, mice were randomized to receive vehicle (sunflower oil; MilliporeSigma, S5007) or 3 mg at day 1 and 2 mg thereafter of tamoxifen (MilliporeSigma, T5648) daily for 3 days, prepared in vehicle. Vehicle or tamoxifen injection was administrated by oral gavage. Mice were monitored every other day for lymphoma development by palpation and euthanized when they reached predefined humane endpoints. Lymph nodes and spleens were collected for further analysis.

### Cell culture

#### Cell lines.

All the human cell lines used in this study were obtained from Cell Services at the Francis Crick Institute. All cell lines underwent routine quality control, which included mycoplasma detection, short tandem repeats (STR) profiling, and species identification for validation. Cells were cultured at 37°C in a humidified atmosphere of 5% CO_2_. Daudi, Ramos, and Namalwa cells were cultured in RPMI 1640 medium (Thermo Fisher Scientific, 21875091) supplemented with 5% FCS; JEKO-1, Raji, DOHH2, and DG-75 cells were cultured in RPMI 1640 medium supplemented with 10% FCS; BJAB and OCI-LY3 cells were culture in RPMI 1640 medium supplemented with 20% FCS; KARPAS 422 and SUDHL4 cells were cultured in ATCC RPMI 1640 medium (Thermo Fisher Scientific, A1049101) supplemented with 20% and 10% FCS, respectively; GRANTA-519 cells were cultured in DMEM (Thermo Fisher Scientific, 41966052) supplemented with 10% FCS and 2 mM L-glutamine; and OCI-LY17 cells were cultured in IMDM (Thermo Fisher Scientific, 21980032) supplemented with 20% FCS.

#### Primary cells.

Buffy cones were obtained from the UK National Blood Service for investigation of healthy B cells. Human naive B cells were isolated using the MACSxpress Whole Blood Naive B Cell Isolation Kits (Miltenyi Biotec, 130-098-186) according to the manufacturer’s instructions. Residual erythrocytes were lysed using 1× Red Blood Cell Lysis Buffer (Invitrogen, 00-433). Cells were then washed in full medium consisting of RPMI 1640 (Thermo Fisher Scientific, 21875091), 10% FCS (Thermo Fisher Scientific, 26400044), 2.5% 1M HEPES buffer (MilliporeSigma, H0887), 1% GlutaMAX (Thermo Fisher Scientific, 35050038), 1% penicillin-streptomycin (Thermo Fisher Scientific, 15140122), and 0.1% 0.05M β-mercaptoethanol (MilliporeSigma, M3148). Cell viability (trypan blue) was > 90%. The purity and recovery of the enriched B cells was evaluated by flow cytometry, and the proportion of CD19^+^ cells was > 95% in all cases.

Resting B cells were stimulated with 20 μg/mL goat F(ab’)2 anti–human IgM + IgG (Stratech Scientific, 109-006-127), 250 ng/mL human recombinant MEGACD40L (Enzo, ALX-522-110), 20 ng/mL Recombinant human IL-4 (R&D system, 204-IL-010), and 7.5 µg/mL CpG-ODN 2006 (Source Bioscience). Cells were collected at specific time points for analysis.

Murine naive B cells were purified from spleen by magnetic negative selection by using a Pan B Cell Isolation Kit (Miltenyi Biotec, 130-095-813) according to the manufacturer’s instructions and maintained in appropriate culture medium. Typically, purified B cells were plated at a concentration of 1 × 10^7^/mL and cultured with at 37°C with 5% CO_2_ in RPMI 1640 medium (Thermo Fisher Scientific, 21875091) supplemented 10% FCS (Thermo Fisher Scientific, 26400044), 2.5% 1M HEPES buffer (MilliporeSigma, H0887), 1% GlutaMAX (Thermo Fisher Scientific, 35050038), 1% penicillin-streptomycin (Thermo Fisher Scientific, 15140122), and 0.1% 0.05M β-mercaptoethanol (MilliporeSigma, M3148). Murine resting B cells were stimulated with 20 μg/mL goat F(ab’)2 anti–mouse IgM + IgG (Stratech Scientific, 115-006-068), 250 ng/mL mouse recombinant MEGACD40L (Enzo, ALX-522-120), and 20 ng/mL recombinant mouse IL-4 (R&D system, 404-ML). Cells were collected at specific time points for analysis. For all serine- and glycine-deprivation experiments, cells were cultured in standard media deprived of L-serine and L-glycine.

### Immunoblot analysis

Cells were lysed on ice for 30 minutes using lysis buffer (1% [vol/vol] Nonidet P-40, 20 mM Tris-HCl [pH 8.0], 150 mM NaCl, and 5 mM EDTA with protease inhibitors [Roche Diagnostics, 11873580001]) and phosphatase inhibitors (sodium fluoride and sodium orthovanadate;MilliporeSigma, S7920 and S6508). Samples were centrifuged (21,000*g*, 15 minutes, 4°C), and the protein content of the supernatant was measured using the Protein Assay Dye Reagent (Bio-Rad, 500-0006). Immunoblotting was performed using from 10 μg to 50 μg of protein lysate. Antibodies were used for detecting the following proteins: PHGDH (Cell Signaling Technology; human specific, 66350; mouse-specific, 13428), PSAT1 (Thermo Fisher Scientific, PA5-22124), PSPH (Thermo Fisher Scientific, PA5-22003), anti-HSC70 (Santa Cruz Biotechnology, sc-7298). All secondary HRP-conjugated antibodies were from Cell Signaling Technology. Membranes were exposed using Amersham Hyperfilm ECL (GE Healthcare) and developed using a Curix 60 developer (Agfa). Films were scanned and quantified using ImageJ 1.50c (NIH). All values were normalized to the HSC70 loading control, and relative fold-change was calculated with the isotype control antibody treated cells taken as 100% of expression.

### qPCR

Total RNA was isolated from cells using the RNeasy Mini-Kit (QIAGEN, 74104) according to the manufacturer’s instructions. RNA was reverse transcribed using oligo(dT) primers (Promega, C1101) and M-MLV (Promega, M1701) in the presence of RNase inhibitor (Promega, N2511). Quantitative PCR (qPCR) was performed on a QuantStudio 12 Flex Real-Time (Applied Biosystems). A standard curve was generated for each human gene from untreated HeLa cells and for each mouse gene from untreated NIH3T3 cells. The average complementary DNA concentration was determined using the standard curve method and made relative to β-actin. Primers used for qPCR were all purchased from Applied Biosystems and are as follow: TaqMan probe for human PHGDH, Hs01106329_m1; TaqMan probe for human PSAT1, Hs00795278_mH; TaqMan probe for human PSPH, Hs00190154_m1; TaqMan probe for β-actin, Hs01060665_g1; TaqMan probe for human PHGDH, Mm01623589_g1; TaqMan probe for human PSAT1, Mm07293542_m1; TaqMan probe for human PSPH, Mm01197775_m1; and TaqMan probe for β-actin, Mm02619580_g1.

### IHC

All tissues were fixed in 10% neutral buffered formalin and were embedded in paraffin. Tissue microarrays (TMAs) of triplicate 1 mm diameter cores were prepared from paraffin blocks using a manual tissue arrayer (Beecher Scientific) as previously described (54). Cut sections or TMAs were fully drained and placed in a 60°C oven for a minimum of 2 hours. Slides were then deparaffinized in xylene and rehydrated using a series of absolute ethanol solutions and distilled water. Heat-induced epitope retrieval (HIER) was performed for 10 minutes in a pressure cooker using boiling citric acid–based antigen unmasking solution (Vector, H3300). After retrieval, slides were placed into a wash buffer containing 1× TBS-tween (Agilent Dako, S3306) before IHC staining. Specific primary antibodies were diluted in Agilent antibody diluent (catalog S080983-2) and incubated for 40 minutes at RT. The dilutions and manufacturer details of all the antibodies used are listed in Table 1. The slides were washed and then incubated with biotinylated secondary antibodies (Vector; anti–rat BA-9401, anti–rabbit BA-1000) for 30 minutes at RT. The slides were washed again before staining with the VECTASTAIN Elite ABC peroxidase kit (Vector, PK-6100), in combination with a DAB substrate kit (Vector, SK-4100). The slides were then counterstained with hematoxylin and then scanned with the Pannoramic 250 Flash II system. Immunostaining was quantified by computerized image analysis using the DensitoQuant tool in Pannoramic Viewer (3DHistTech) to generate H-scores for each area of tissue. This application identifies the positive stain, based on an automatic color separation method, through which individual positive pixels are counted and classified based on intensity and threshold ranges. The positive pixels are further classified into high (3+), medium (2+), or low (1+) based on the biomarker signal intensity. The H-score is given by the ratio of the weighted sum of the number of positive pixels to the total number of pixels to capture both the intensity and the extent of staining of the biomarker of interest (55).

### Flow cytometry

For surface staining, single-cell suspension was washed twice in staining buffer (PBS containing 2% FCS and 2 mM EDTA). To detect sheep RBC–induced GC, DZ, and LZ B cells, splenocytes were incubated with directly conjugated monoclonal antibodies (anti–CD19-PE [BD Bioscience, 7073631], anti–B220-BV510 [BioLegend, 103247], anti–FAS-BV421 [BD Bioscience, 562633], anti–CD38-PE-Cy7 [BioLegend, 102718], anti–CXCR4-Alexa488 [eBioscience, 53-9991-80], anti–CD86-APC [BioLegend, 105012]) and Fixable viability dye eFluor780 staining (eBioscience, 65-0865-14) for 30 minutes on ice protected from light. Cells were then washed and resuspended in staining buffer and kept at 4°C until analysis. To identify NP-specific plasma cells following NP-CGG immunization, splenocytes were incubated with directly conjugated monoclonal antibodies (anti–CD19-BUV395 [BD Horizon, 563557], anti–B220-BV510 [BioLegend, 103247], anti–FAS-PerC/P-Cy5.5 [eBioscience, 45-5892-82], anti–CD38-PE-Cy7 [BioLegend, 102718], anti–IgM-APC [eBioscience, 17-5790-82], anti–IgG1-BV421 [BD Horizon, 562580], anti–CD138-BV786 [BD Bioscience, 740880], and anti–NP-PE [generated in-house]). To identify B cell populations in BM, cells were incubated with the following combination of monoclonal antibodies: anti–IgD-FITC (eBioscience, 11-5993-82), anti–CD138-PE (BioLegend, 142504), anti–CD2-PE-Cy7 (BioLegend, 100114), anti–IgM-APC (eBioscience, 17-5790-82), and anti–B220-BV510 (BioLegend, 103247). To identify splenic B cell populations, cells were incubated with directly conjugated monoclonal antibodies: anti–CD93-FITC (BioLegend, 136508), anti–CD138-PE (BioLegend, 142504), anti–CD23-PE-Cy7 (BioLegend, 740414), anti–IgM-APC (eBioscience, 17-5790-82), anti–B220-BV510 (BioLegend, 103247).

To assess PHGDH and PSAT1 levels in vivo, splenocytes were first surface stained for GC markers and then washed twice in staining buffer and treated with fixation/permeabilization solution Cytofix/Cytoperm (BD Bioscience, 554722) for 30 minutes on ice in the dark. Cells were washed twice in 1× Perm/Wash buffer (BD Bioscience, 554723) and then incubated with either anti-PHGDH (1/250) (Cell Signaling Technology, 13428) or anti-PSAT1 (1/400) (Thermo Fisher Scientific, PA5-22124) in staining buffer for 1 hour on ice in the dark and washed twice as previously. Cell were stained with the secondary antibody anti–rabbit Alexa Fluor 647 (1/500) (Cell Signaling Technology, 4414) for 30 minutes at RT. Cells were then washed, resuspended in staining buffer, and kept at 4°C until analysis. For intracellular active Caspase-3 staining, cells were stained for surface markers (when required) and then fixed/permeabilized by using PE Active Caspase-3 Apoptosis Kit (BD Bioscience, 550914) according to the manufacturer’s protocol. To evaluate cell cycle profile, 10 μM BrdU was then added to culture media for 30–45 minutes. Cells were then harvested, fixed, and stained with APC anti-BrdU antibody and 7-AAD using the APC BrdU Flow kit (BD Pharmingen, 552598) following the manufacturer’s instructions. Fluorescence was acquired using Fortessa flow cytometer (BD Bioscience) with subsequent analysis using FlowJo software (Tree Star Inc.). Analysis was performed after gating on live singlet cells.

### ELISA

Serial dilutions of serum samples were analyzed by ELISA on NP_2_-BSA–coupled (5 μg/mL) microtiter plates to detect high-affinity NP–specific IgG1. Alkaline phosphatase–conjugated (AP-conjugated) primary antibodies anti-IgG1 (9054-01, Southern Biotech) were developed with p-nitrophenyl phosphate dissolved in Tris buffer (MilliporeSigma). The absorbance was measured at 405 nm and plotted against dilution, and relative antibody titres were read as the dilution where absorbance reached an arbitrary threshold.

### Metabolite extraction and LC-MS

Cells were seeded at 5 × 10^6^ per well of serine/glycine-free complete media for 1 hour. After 1 hour, cells were transferred to serine/glycine-free media containing ^13^C-U-glucose (2 g/L) (Cambridge Isotope Laboratories, CLM-1396-PK) for an additional 2 hours. Cells were then washed with PBS before being metabolically quenched by transferring to dry ice. For PHGDH inhibition experiments, cells were pretreated with 10 μM PH-755 (Raze Therapeutics) diluted in DMSO or DMSO alone for 1 hour before labeling with ^13^C-U-glucose. Metabolites were extracted by resuspending the cell pellet in ice-cold HPLC-grade methanol, acetonitrile, and H_2_O at a volume ratio 50:30:20 for 1 hour at 4°C with 3 sonication steps (8 minutes each step) within the hour. After sonication, samples were centrifuged for 10 minutes at 21,000*g* (4°C), and the supernatant was collected. The extraction solvent was dried in a glass insert placed in a LC-MS vial (Agilent, 5182-0716) using a SpeedVac (Christ RVC 2-33 CDplus). Then, 20 μL of metabolite extraction buffer containing 5 μM ^13^C,^15^N-Valine (used as an internal extraction standard) were added to each dried sample, and metabolite samples were stored at −80°C for subsequent analyses. Triplicates of identically seeded and treated cells were analyzed.

Metabolite analysis was performed by LC-MS using a Q-Exactive Plus (Orbitrap) mass spectrometer (Thermo Fisher Scientific) coupled with a Vanquish UHPLC system (Thermo Fisher Scientific). The chromatographic separation was performed on a SeQuant Zic pHILIC (Merck Millipore) column (5 μm particle size, polymeric, 150 × 4.6 mm) using a gradient program at a constant flow rate of 300 μL/min over a total run time of 25 minutes. The elution gradient was programmed as a decreasing percentage of solvent B from 80% to 5% during 17 minutes, holding at 5% of B during 3 minutes, and finally reequilibrating the column at 80% of B during 4 minutes. Solvent A was 20 mM ammonium carbonate solution in water supplemented by 4 mL/L of a solution of ammonium hydroxide at 35% in water, and solvent B was acetonitrile. MS was performed with positive/negative polarity switching using a Q Exactive Orbitrap (Thermo Fisher Scientific) with a HESI II probe. MS parameters were as follows: spray voltage 3.5 and 3.2 kV for positive and negative modes, respectively; probe temperature 320°C; observe sheath and auxiliary gases at 30 and 5 arbitrary units, respectively; and observe full scan range at 70–1,050 *m/z*, with settings of AGC target and resolution as balanced and high (3 × 10^6^ and 70,000), respectively. Data were recorded using Xcalibur 4.2.47 software (Thermo Fisher Scientific). Mass calibration was performed for both ESI polarities before analysis using the standard Thermo Fisher Scientific Calmix solution. To enhance calibration stability, lock-mass correction was also applied to each analytical run using ubiquitous low-mass contaminants. Parallel reaction monitoring (PRM) acquisition parameters were as follows: resolution was 17,500, and collision energies were set individually in high-energy collisional dissociation (HCD) mode. Metabolites were identified and quantified by accurate mass and retention time and by comparison with the retention times, mass spectra, and responses of known amounts of authentic standards using TraceFinder 4.1 EFS software (Thermo Fisher Scientific). Label incorporation and abundance was estimated using TraceFinder 4.1 EFS software. The level of labeling of individual metabolites was estimated as the percentage of the metabolite pool containing 1 or more ^13^C atoms after correction for natural abundance isotopes. Abundance was given relative to the internal standard.

### Single-cell RNA-Seq analysis of human tonsillar B cell subsets

B cell populations from previously published tonsillar immune single-cell RNA-Seq (37) were identified by using unbiased clustering of gene expression and analysis of their antibody repertoires (i.e., isotype frequencies, somatic hypermutation levels, and clonal expansion) using Seurat (v3) (56). Gene expression markers used to define the groups presented here include the following: naive (*TXNIP*, *FCER2*, *FCMR*), activated (*EGR1*, *CD69*, *JUN*), pre-GC (*MIR155HG*, *BHLHE40*, *PSME2*, *CCND2*), memory (*TNFRSF13B*, *CD44*, *VIM*, *FCRL4*), GC (*BCL6*, *CD38*, *EZR*, *SERPINA9*, *LMO2*), cycling GC (*AURKB*, *MKI67*, *UBE2C*), and plasmablast (*XBP1*, *PRDM1*, *MZB1*). Gene expression counts were imputed for visualization using MAGIC (57).

### Statistics

Statistical significance was assessed Prism 9.1.1 software (GraphPad Software). Groups were compared using a 2-tailed unpaired *t* test, Mann-Whitney *U* test, or 1-way ANOVA with Tukey’s post hoc test, depending on the number of groups and distribution. For survival comparison, a log-rank test was used. Data are shown as the mean ± SEM. A *P* value of less than or equal to 0.05 was considered to indicate statistical significance. When analyzing variables with more than 2 categories, *P* values were adjusted for multiple comparisons.

### Study approval

All animal studies were conducted in compliance with UK Home Office approved licences (Animals [Scientific Procedures] Act 1986 and the EU Directive 2010). Animal experiments were subject to ethical review by the Francis Crick Animal Welfare and Ethical Review Body and carried out under UK Home Office project licence P319AE968. Lymphoma samples were obtained from Barts Cancer Institute tissue bank (London, United Kingdom). Ethical approval was confirmed by the East London & The City Health Authority Local Research Ethics Committee (no. 10/H0704/65), and written informed consent was also obtained in accordance with the Declaration of Helsinki.

## Author contributions

AD designed and performed the experiments, analyzed and interpreted data, and wrote the manuscript; MT and ECC designed experiments and analyzed and interpreted data; NL and JIM designed and performed the metabolomic experiments, as well as analyzed and interpreted the LC-MS data; RLS, KK, and AJC designed, performed, and analyzed the IHC experiments; HWK and LKJ analyzed and interpreted the single-cell RNA-Seq data sets; PC analyzed and interpreted the statistical data; ASG and LZ designed the flow cytometry and ELISA experiments, and they analyzed and interpreted data; JGG and DPC designed experiments and edited the manuscript; KHV designed experiments, analyzed and interpreted the data, edited the manuscript, and supervised the study; and JCR designed and performed the experiments, analyzed and interpreted the data, wrote and edited the manuscript, and supervised the study. All authors approved the final submission.

## Supplementary Material

Supplemental data

## Figures and Tables

**Table 1:**
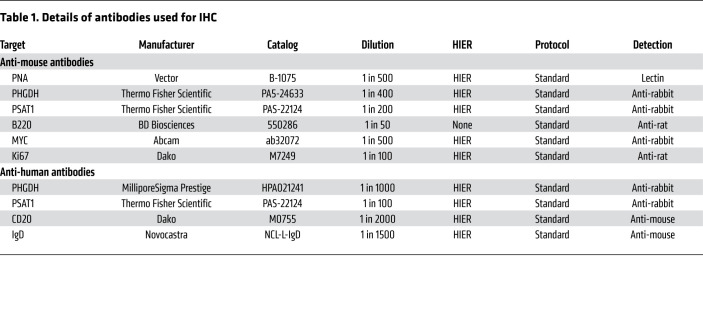
Details of antibodies used for IHC

## Version 1 03/22/2022

In-Press Preview

## Version 2 05/02/2022

Electronic publication
