# Supplementary material for: PHGDH is required for germinal center formation and is a therapeutic target in MYC-driven lymphoma
Source: J Clin Invest. 2022 May 2;132(9):e153436. doi: 10.1172/JCI153436 (PMC9057607; doi:10.1172/JCI153436)
Supplement: Supplemental data [file jci-132-153436-s164.pdf]

Supplementary data for:

PHGDH is required for germinal center formation and is a therapeutic target in *MYC*-driven lymphoma

Annalisa D'Avola[1], Nathalie Legrave[1], Mylène Tajan[1], Probir Chakravarty[1], Ryan L. Shearer[2], Hamish W. King[3], Katarina Kluckova[2], Eric C. Cheung[1], Andrew J. Clear[2], Arief Gunawan[1], Lingling Zhang[1], Louisa K. James[3], James I. MacRae[4], John G. Gribben[2], Dinis P. Calado[1], Karen H. Vousden[1], John C. Riches[1,2]

[1]The Francis Crick Institute, 1 Midland Road, London, NW1 1AT, United Kingdom

[2]Centre for Haemato-Oncology, Barts Cancer Institute, Queen Mary University of London, 3rd Floor John Vane Science Centre, Charterhouse Square, London EC1M 6BQ, United Kingdom

[3]Centre for Immunobiology, Blizard Institute, Queen Mary University of London, London, UK

[4]Metabolomics Science Technology Platform, The Francis Crick Institute, 1 Midland Road, London, NW1 1AT, U.K

SUPPLEMENTARY FIGURES

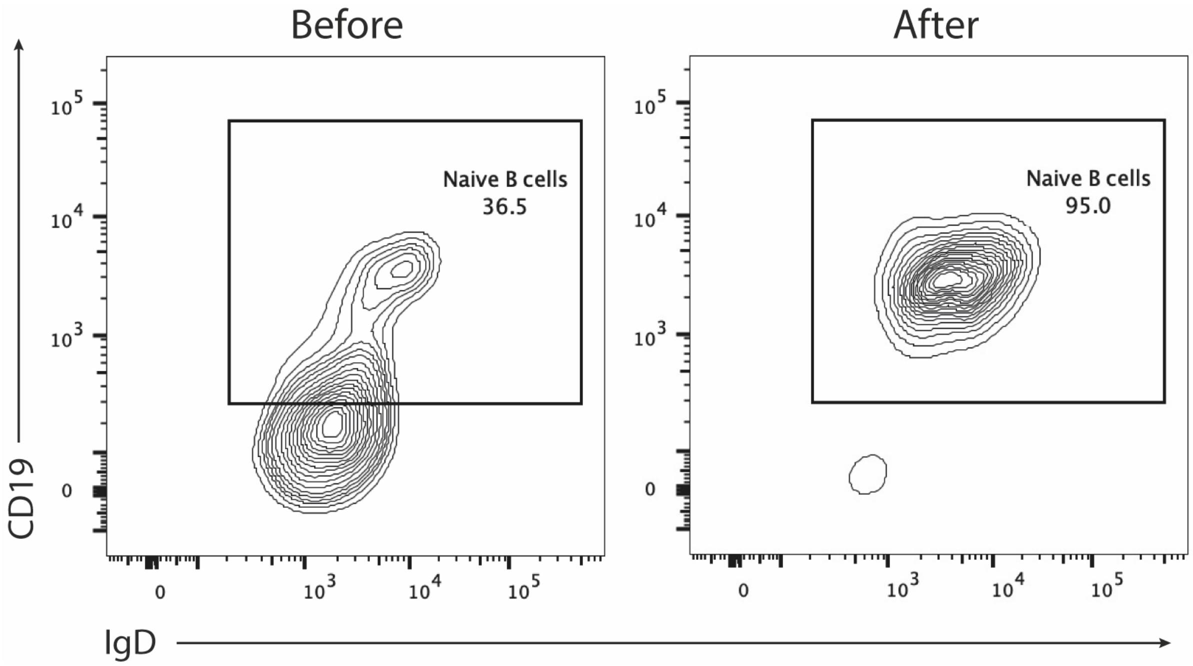

Supplementary Figure 1. Isolation of human naïve B cells

Untouched naïve B cells were isolated from 30 mL of human EDTA-anticoagulated whole blood using the MACSxpress® Whole Blood Naive B Cell Isolation Kit, a MACSmix™ Tube Rotator, and a MACSxpress Separator. The isolated cells were fluorescently stained with anti-CD19 and anti-IgD antibodies and analyzed by flow cytometry. Cell debris, non-leukocytes, and dead cells were excluded from the analysis based on CD45 expression, scatter signals, and live/dead dye.

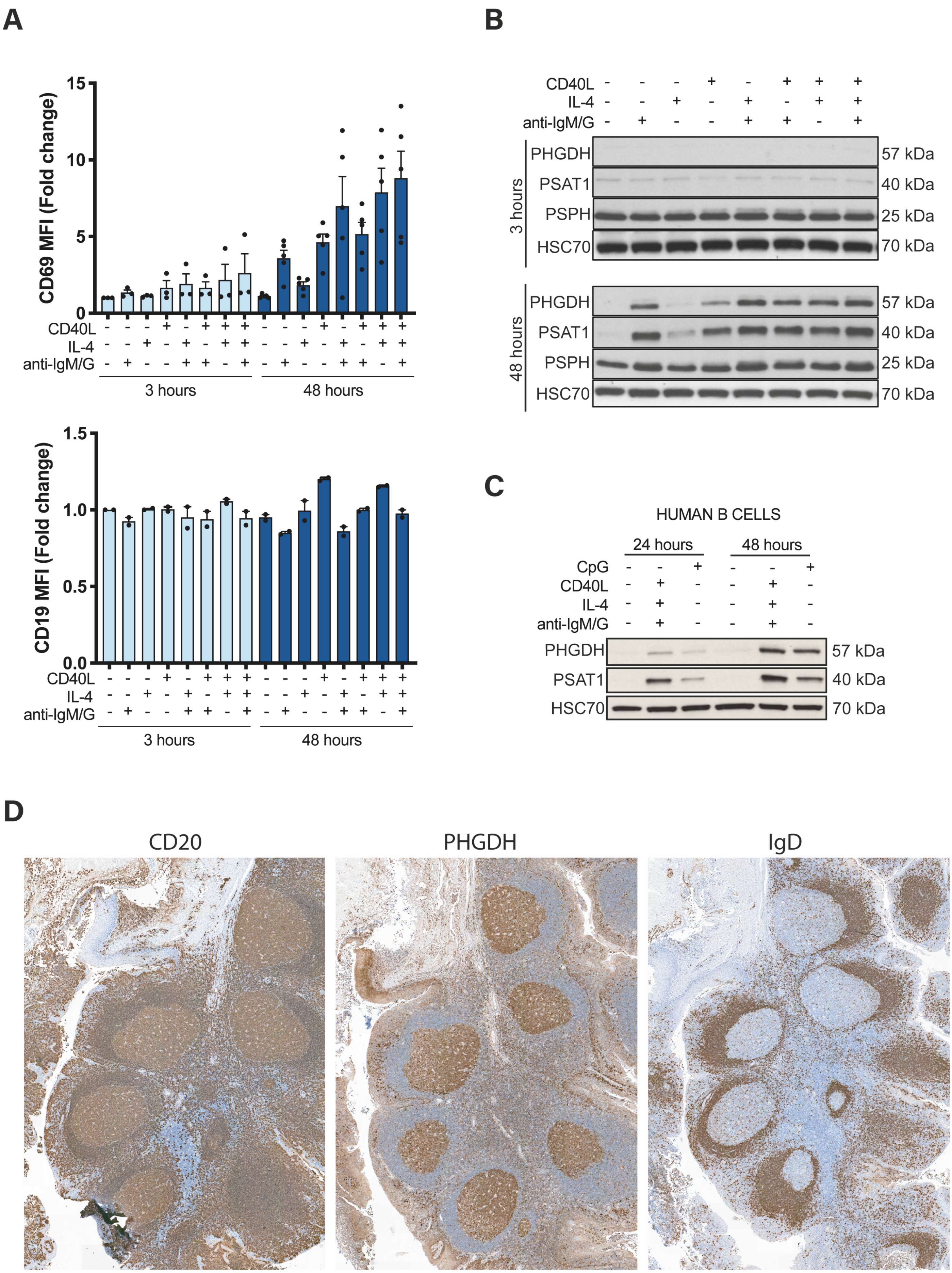
A
B
C
D
CD69 MFI (Fold change)
CD19 MFI (Fold change)
CD40L
IL-4
anti-IgM/G
3 hours
48 hours
PHGDH
PSAT1
PSPH
HSC70
57 kDa
40 kDa
25 kDa
70 kDa
HUMAN B CELLS
24 hours
CpG
CD20
IgD

Supplementary Figure 2. Activation of human B cells leads to upregulation of the serine synthesis pathway.

(**A**) Flow cytometric analysis of surface expression of CD69 (above) and CD19 (below) and (**B**) representative immunoblots of PHGDH, PSAT1 and PSPH proteins levels in resting human mature naïve B cells (-) or in B cells stimulated with anti-IgM/G, CD40L and or IL-4 for 3 and 48 hours (+). (**C**) Representative immunoblots of PHGDH, PSAT1 proteins levels in resting human mature naïve B cells (-) or in B cells stimulated (+) with either anti-IgM/G/CD40L/IL-4 or CpG-ODN for 24 and 48 hours. (**D**) Representative immunohistochemical staining for CD20, PHGDH and IgD in human tonsils from patients after tonsillectomy. $CD20^{+}IgD^{+}$ mantle zone B cells outside of the GC express low levels of PHGDH whereas $CD20^{+}IgD^{-}$ GC B cells strongly express PHGDH. Individual samples (dots) and means (bars) values are plotted.

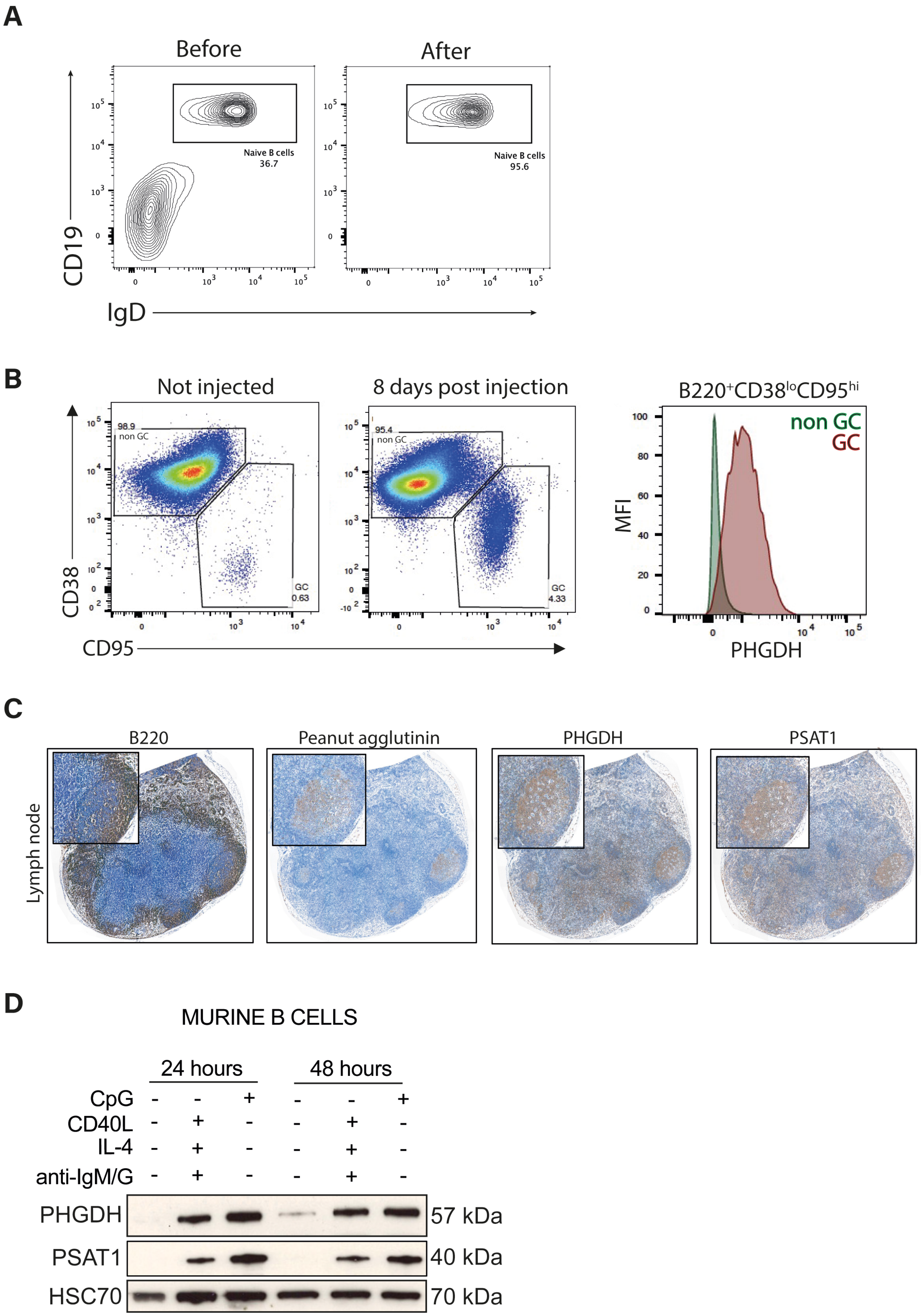
A
Before
After
CD19
IgD
Naive B cells
36.7
Naive B cells
95.6
B
Not injected
8 days post injection
B220+CD38loCD95hi
98.9
non GC
95.4
non GC
GC
0.63
GC
4.33
CD38
CD95
MFI
PHGDH
non GC
GC
C
B220
Peanut agglutinin
PHGDH
PSAT1
Lymph node
D
MURINE B CELLS
24 hours
48 hours
CpG
CD40L
IL-4
anti-IgM/G
PHGDH
PSAT1
HSC70
57 kDa
40 kDa
70 kDa

Supplementary Figure 3. In vivo activation of serine synthesis pathway in GC B cells

(**A**) Untouched naïve B cells were isolated from spleens from wild type mice. The cells were fluorescently stained with anti-CD19-PE and anti-IgD and analyzed by flow cytometry. Cell debris and dead cells were excluded from the analysis based on scatter signals and live/dead dye. (**B**) Representative flow cytometric analysis of PHGDH expression within GC B cells ($CD19^{+}B220^{+}CD38^{lo}CD95^{hi}$) isolated from BL6 mice immunized with sheep RBCs for 8 days. (**C**) Representative immunohistochemical staining for peanut agglutinin (PNA) as GC marker, PHGDH and PSAT1 on consecutive sections derived from mice lymph nodes 8 days after sheep RBC immunization. Data are representative of three independent experiments. (**D**) Representative immunoblots of PHGDH, PSAT1 proteins levels in resting isolated mouse naïve B cells (-) or in B cells stimulated (+) with either anti-IgM/G/CD40L/IL-4 or CpG-ODN for 24 and 48 hours.

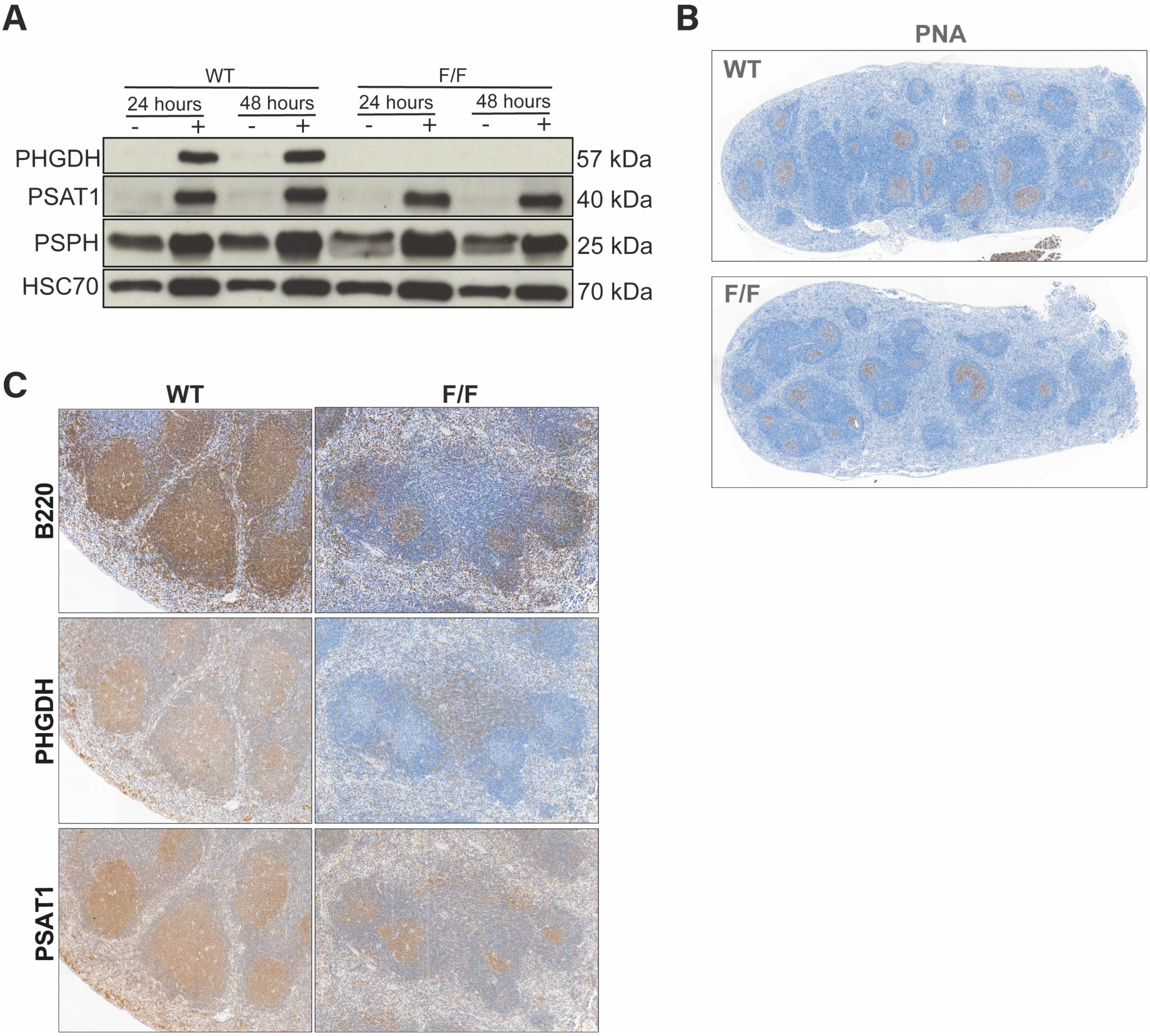

Supplementary Figure 4. Inhibition of PHGDH impairs GC responses

(**A**) Analysis of PHGDH, PSAT1 and PSPH protein levels in B220^{+} B cells isolated from either *PHGDH*$^{+/+}$*/CD19-Cre* (WT) or *PHGDH*$^{fl/fl}$*/CD19-Cre* (F/F) immunized with sheep BC for 8 days, and treated in vitro with (+) or without (-) anti-IgM antibody, CD40 ligand (CD40L) and interleukin-4 (IL-4) for 24 and 48 hours before protein extraction. (**B**) Representative immunohistochemical staining for PNA in spleen sections derived from *Phgdh*$^{+/+}$*;Cd19-Cre* (WT) and *Phgdh*$^{fl/fl}$*;Cd19-Cre* (F/F) mice 8 days after immunization with sheep RBCs. Data are representative of three independent experiments. (**C**) Representative immunohistochemical staining for B220, PHGDH and PSAT1 on consecutive spleen sections derived from mice described in (**B**).

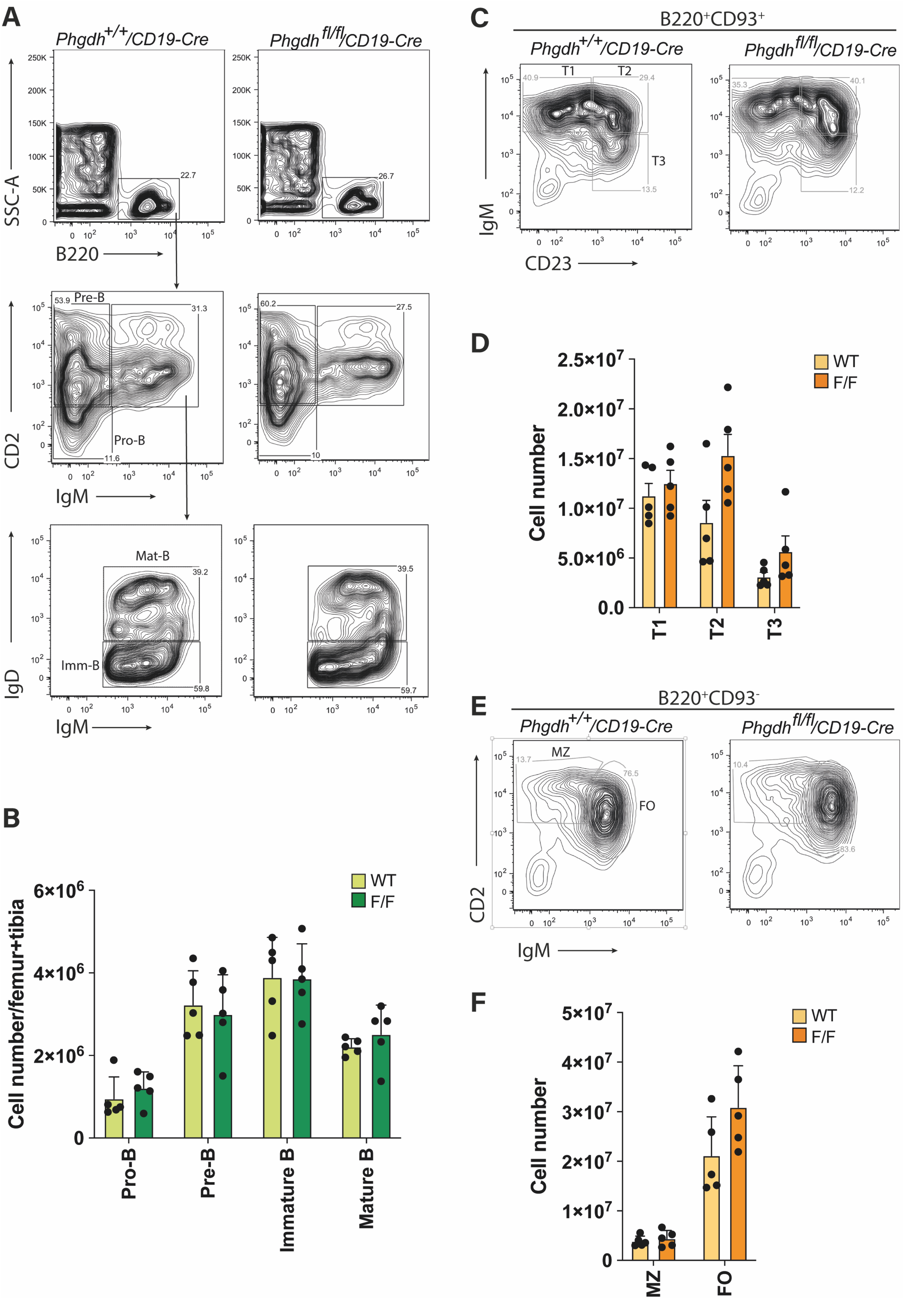

A
Phgdh+/+/CD19-Cre
Phgdhfl/fl/CD19-Cre
SSC-A
B220
22.7
26.7
CD2
IgM
53.9
Pre-B
31.3
Pro-B
11.6
60.2
27.5
10
IgD
Mat-B
39.2
Imm-B
59.8
39.5
59.7
B
Cell number/femur+tibia
WT
F/F
Pro-B
Pre-B
Immature B
Mature B
C
B220+CD93+
T1
T2
T3
40.9
29.4
13.5
35.3
40.1
12.2
CD23
D
Cell number
T1
T2
T3
E
B220+CD93-
MZ
FO
13.7
76.5
10.4
83.6
F
MZ
FO

Supplementary Figure 5. Analysis of total bone marrow and splenic B-cell populations in *Phgdh*$^{+/+}$/*CD19-Cre* and *Phgdh*$^{fl/fl}$/*CD19-Cre* littermates.

(**A**) Flow cytometry gating strategy to identify different B-cell populations in bone marrow of *Phgdh*$^{fl/fl}$/*CD19-Cre* mice. Arrows indicate flow of analysis. The same strategy was used for *Phgdh*$^{+/+}$/*CD19-Cre* littermates. Pro-B cells are defined as B220^{+}CD2^{low}IgM^{-}, pre-B cells as B220^{+}CD2^{high}IgM^{-}, immature-B cells as B220^{+}CD2^{high}IgM^{+}IgD^{low} and mature-B cells as B220^{+}CD2^{high}IgM^{+}IgD^{high} (**B**) Numbers of different B-cell populations in bone marrow *Phgdh*$^{+/+}$/*CD19-Cre* and *Phgdh*$^{fl/fl}$/*CD19-Cre* mice as defined in (**A**). (**C** and **E**) Representative flow cytometry analysis to identify transitional (T) B cells (T1: B220^{+}CD93^{high}IgM^{high}CD23^{low}; T2: B220^{+}CD93^{high}IgM^{high}CD23^{high}; T3: B220^{+}CD93^{high}IgM^{int}CD23^{high}), follicular (FO: B220^{+}CD93^{low}IgM^{high}CD2^{int}) and marginal zone B cells (MZ: B220^{+}CD93^{low}IgM^{low}CD2^{high}) in spleen of *Phgdh*$^{+/+}$/*CD19-Cre* and *Phgdh*$^{fl/fl}$/*CD19-Cre* mice. (**D** and **F**) Numbers of different B-cell populations in spleen of *Phgdh*$^{+/+}$/*CD19-Cre* and *Phgdh*$^{fl/fl}$/*CD19-Cre* mice littermates as defined in (**C** and **E**). Numbers indicate the percentage of cells in each gate, relative to total erythrocyte-depleted splenocytes. Data are representative of five independent experiments Individual samples (dots) and means (bars) values are plotted.

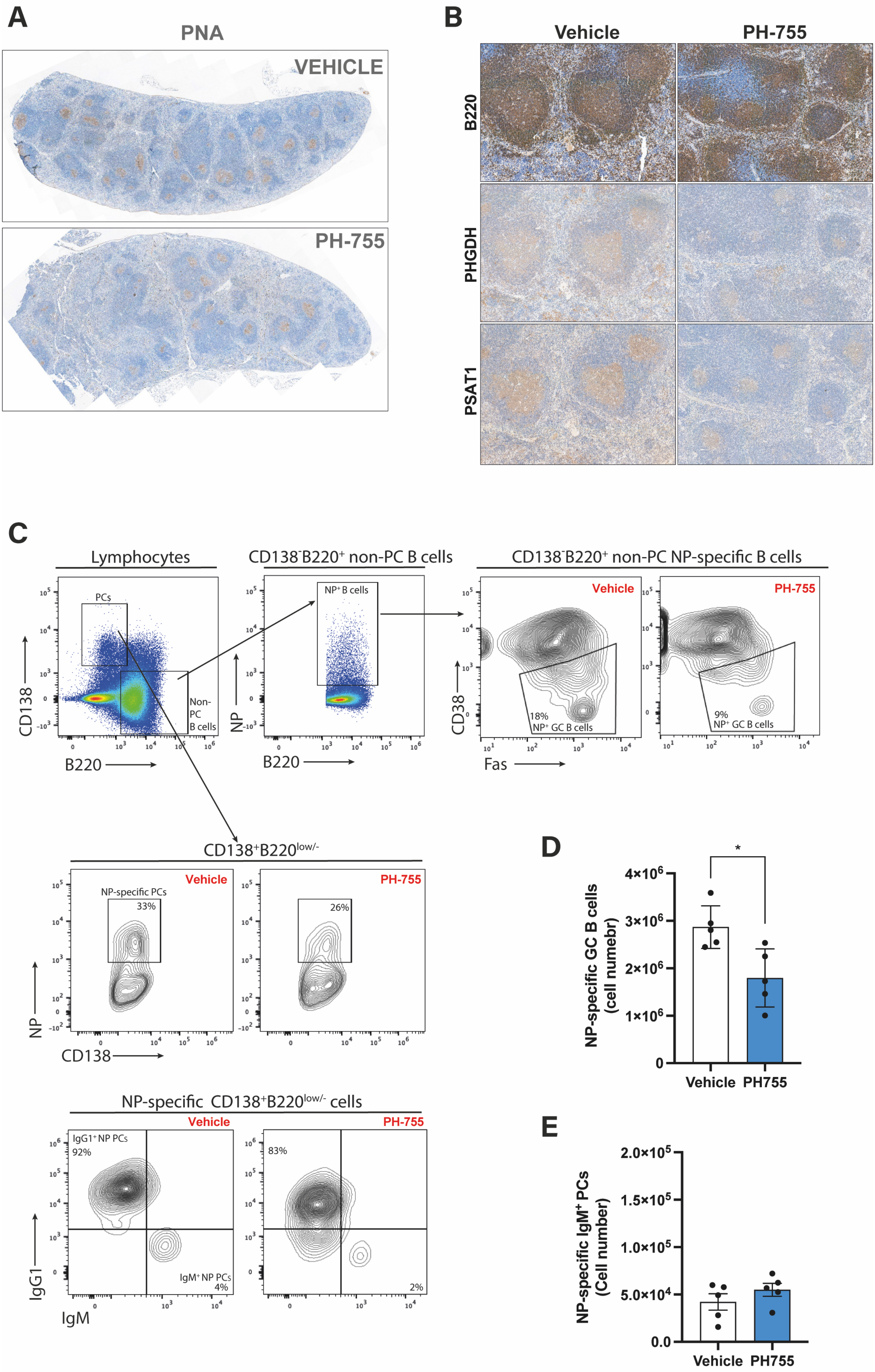
A
PNA
VEHICLE
PH-755
B
Vehicle
PH-755
B220
PHGDH
PSAT1
C
Lymphocytes
CD138-B220+ non-PC B cells
CD138-B220+ non-PC NP-specific B cells
PCs
Non-PC B cells
NP+ B cells
Vehicle
PH-755
18%
NP+ GC B cells
9%
NP+ GC B cells
CD138
B220
NP
CD38
Fas
CD138+B220low/-
NP-specific PCs
33%
26%
NP
CD138
NP-specific CD138+B220low/- cells
IgG1+ NP PCs
92%
83%
IgM+ NP PCs
4%
2%
IgG1
IgM
D
NP-specific GC B cells (cell numebr)
4×10^6
3×10^6
2×10^6
1×10^6
0
*
Vehicle
PH755
E
NP-specific IgM+ PCs (Cell number)
2.0×10^5
1.5×10^5
1.0×10^5
5.0×10^4
0.0
Vehicle
PH755

Supplementary Figure 6. PHGDH inhibition impaired GC B cell response and plasma cell differentiation.

(**A**) Mice were injected with sheep RBCs 24 hours before starting treatment with PHGDH inhibitor PH-755 or vehicle control. Representative immunohistochemical staining for PNA in spleen sections from mice injected with sheep RBC and treated with PH-755 or vehicle control. Data are representative of three independent experiments. (**B**) Representative immunohistochemical staining for B220, PHGDH and PSAT1 on consecutive spleen sections derived from mice described in (**A**). Data are representative of three independent experiments. (**C**) Animals were injected with NP-CGG one day before the beginning of PH-755 treatment. Popliteal lymph nodes were collected 8 days post NP-CGG immunization. NP-specific GC responses and NP-specific PC differentiation were assessed by flow cytometry. Figure shows the gate setting of T-D immune response after NP-CGG immunization in presence of vehicle or PH-755. Gates for plasma cells (PCs), NP-specific B cells, NP-specific GC B cells and NP-specific plasma cells are displayed. (**D**) Numbers of NP-specific GC B cells and (**E**) NP-specific IgM+ PCs (total number per popliteal lymph nodes). Wild-type BL6 mice were treated with either Vehicle (n=5) or PH-755 (n=5) for 7 days.

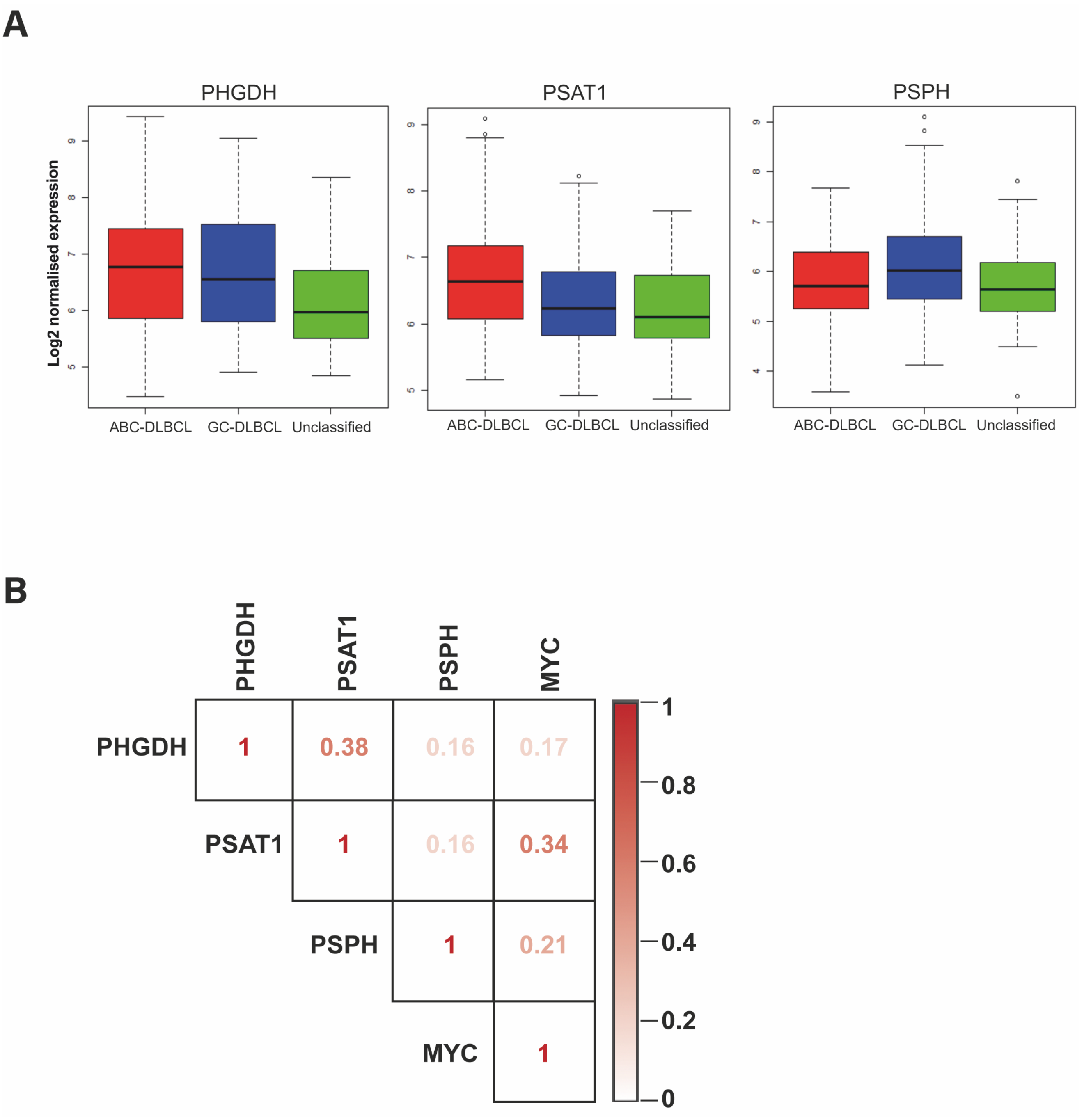

Supplementary Figure 7. Correlation of expression of SSP enzymes with DLBCL subtypes

(**A**) The expression of PHGDH, PSAT1, PSPH mRNA was analyzed in a published dataset (GSE10846) to compare the expression of these enzymes in activated B-cell (ABC) like, germinal centre B-cell (GCB) like, or unclassified DLBCL. (**B**) Analysis of a published dataset (GSE10846) to correlate the gene expression of the serine synthesis pathway enzymes with MYC in DLBCL using the Spearman's correlation coefficient. Graph shows weak positive correlations between MYC expression and PHDGH (r = 0.17), PSAT1 (r = 0.34) and PSPH (r = 0.21).

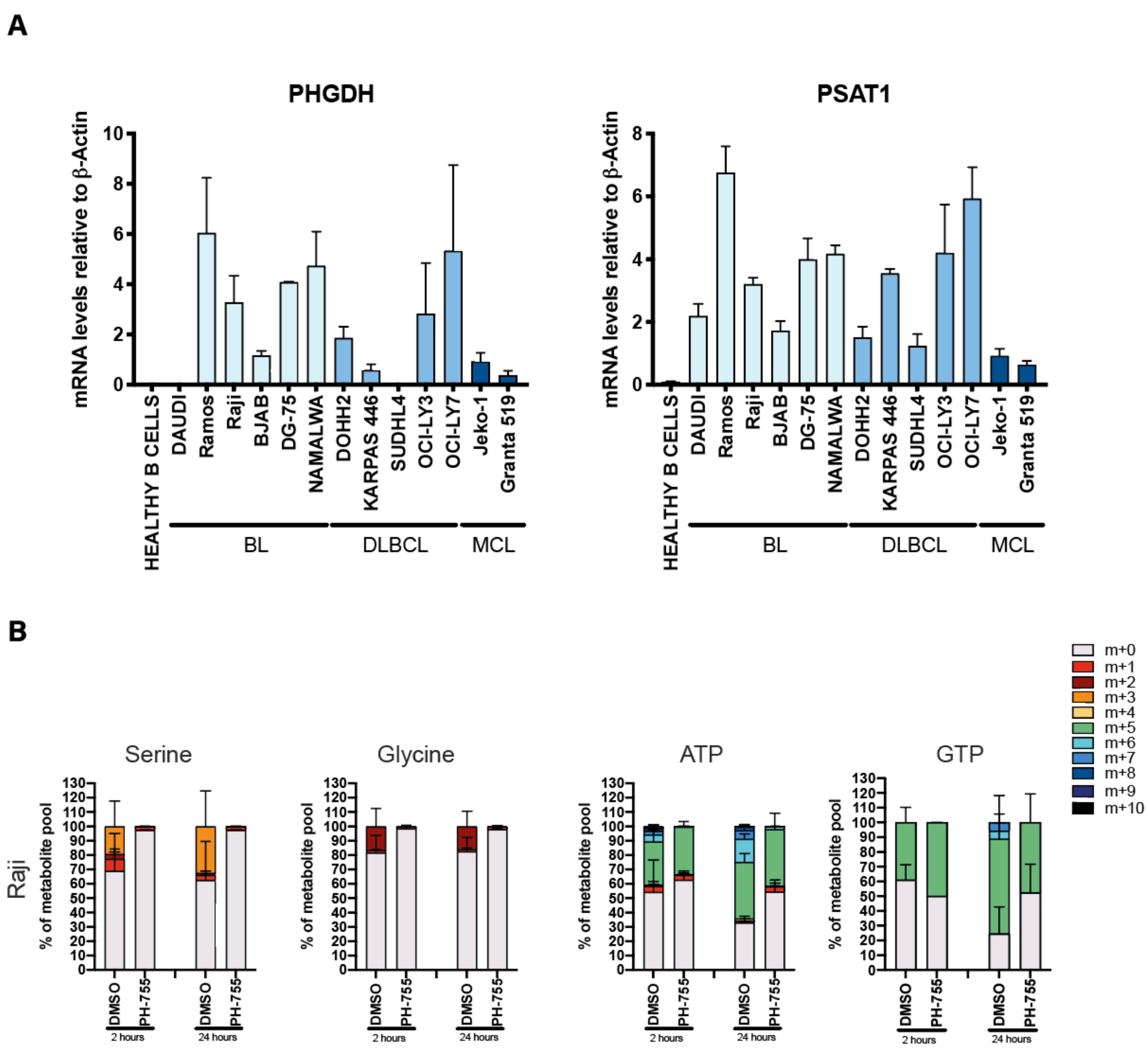

Supplementary Figure 8. Serine synthesis pathway is activated in in Burkitt lymphoma cells (**A**) Relative mRNA expression of SSP enzyme genes from different human B-cell lymphoma lines compared with primary B cells. Cell line were grouped into three groups (BL, DLBCL and mantle cell lymphoma (MCL) cell lines). Data are presented as mean (± SEM) of two independent experiments. (**B**) Mass isotopologue distribution of U-[$^{13}C_6$]-glucose-derived serine, glycine, ATP and GTP for Raji cells cultured for 2 and 24 hours in medium lacking serine and glycine in presence of U-[$^{13}C_6$]-glucose (10mM) and treated with DMSO or 10µM PH-755. Serine, Glycine, ATP and GTP levels were measured by LC-MS. The percentage distribution of each isotopologue for their respective metabolite pool is shown. Data are presented as mean (± SEM) of six repeats and are representative of three independent experiments.

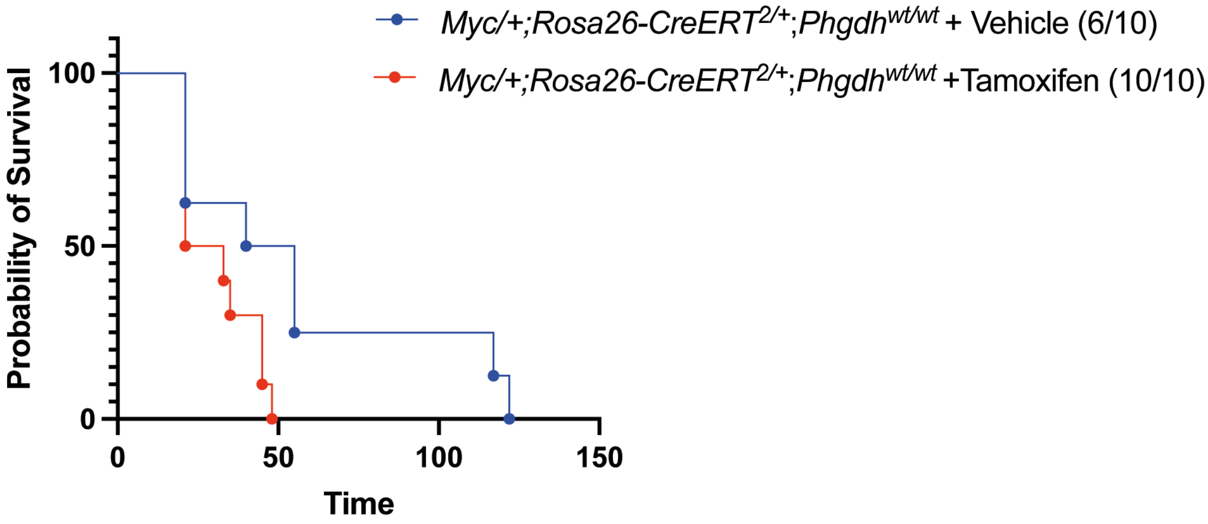

Supplementary Figure 9. Control experiments for impact of Cre-activation after transplantation of lymphoma cells from *Myc/+;Rosa26-CreER$^{T2/+}$;Phgdh$^{+/+}$* mice

Lymphoma cells from *Myc/+;Rosa26-CreER$^{T2/+}$;Phgdh$^{+/+}$* mice were purified and transplanted by intravenous injection into C57BL/6 syngenic mice before the animals were treated with either tamoxifen or vehicle control for 5 days, n=10. Kaplan-Meier analysis for lymphoma-free survival showed no evidence of prolonged survival of mice due to Cre-toxicity to the lymphoma cells after tamoxifen treatment (two-tailed log-rank test).
